# Single-Layer and Stack Dielectric Elastomer Actuators Using Polysiloxanes Modified with Ethylsulfonyl Groups

**DOI:** 10.1021/acsami.5c06610

**Published:** 2025-06-17

**Authors:** Cansu Zeytun Karaman, Thulasinath Raman Venkatesan, Frank A. Nüesch, Dorina M. Opris

**Affiliations:** † Laboratory for Functional Polymers, 28501 Swiss Federal Laboratories for Materials Science and Technology Empa, Ueberlandstr. 129, CH-8600 Dübendorf, Switzerland; ‡ Institute of Chemical Sciences and Engineering, Ecole Polytechnique Federale de Lausanne, EPFL, Station 6, CH-1015 Lausanne, Switzerland; § Department of Materials, Eidgenössische Technische Hochschule Zürich ETHZ, CH-8092 Zurich, Switzerland

**Keywords:** artificial muscles, actuators, stacks, dielectric elastomers, polar polysiloxanes

## Abstract

Dielectric elastomer actuators (DEAs) are soft transducers well-suited to precise motion applications in robotics and prosthetics. However, low dielectric permittivity or very soft elastomers result in a high operating voltage or low force output. These issues can be mitigated using high dielectric permittivity elastomers in a stack actuator. To optimize electromechanical performance, we synthesized high-permittivity polysiloxanes with varying ratios of ethyl sulfonyl thioether and butane thioether groups. The best material exhibited a dielectric permittivity of 16.2 at 10 kHz and 25 °C, a low conductivity of 1.8 × 10^–10^ S cm^–1^, and a large lateral actuation strain of 13% at a low electric field of 8.2 V μm^–1^ (1 Hz, 900 V), whereas state-of-the-art nitrile- and methyl sulfonyl-functionalized polysiloxane required electric fields exceeding 20 V μm^–1^ for the same actuation. A stack of five single-layer actuators using this material as the dielectric exhibited a thickness strain of 4.5% at a low electric field of 14.5 V μm^–1^ (1 Hz, 1600 V). The stack actuator showed stable performance at 1200 V over various frequencies, including 5 and 10 Hz, and maintained a reversible actuation over 4000 cycles at 1 Hz.

## Graphical abstract

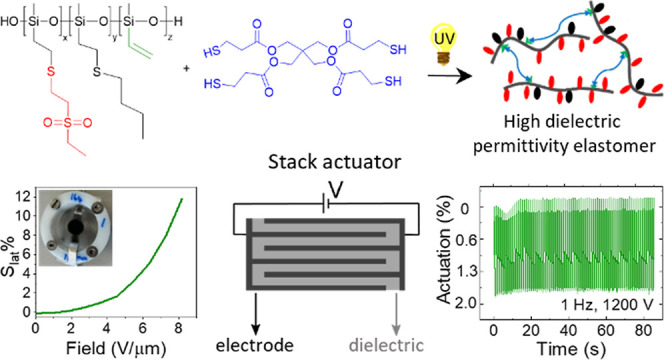

## 1 Introduction

Dielectric elastomers (DEs) have emerged as one of the most promising materials among soft actuators due to their distinctive properties, which combine large deformation, mechanical flexibility, high energy density, voltage-controlled actuation, and operation over a wide temperature and frequency range.^1–6^ These features have placed DEs as crucial components in cutting-edge technologies, including soft robotics, haptic devices, and adaptive optics.^7–12^ However, achieving high-performance DE has always brought great challenges due to the fulfillment of low glass-transition temperature (*T*_g_), excellent mechanical properties, and high dielectric permittivity simultaneously.^12,13^ Polysiloxanes are among the most widely used materials due to their outstanding properties, including a low *T*_g_, excellent stretchability for large deformations, and the ability to tailor their characteristics through synthetic modifications.^13,14^ Despite their many advantages, the primary limitation of polysiloxanes lies in their inherently low dielectric permittivity, which restricts their use in high-performance dielectric elastomer applications.^15,16^ Two approaches have been employed to improve this critical property by incorporating highly polarizable fillers and chemically modifying the elastomer with polar groups.^17–21^ Functionalizing polysiloxanes with polar groups has become a more effective strategy to enhance their dielectric properties.^4,22,23^ Various polar groups have been used to chemically modify the polysiloxane, including nitrile with ε′ = 17.4, chloromethyl with ε′ = 6.8, trifluoro propyl with ε′ = 6.4, and amide with ε′ = 21.^24–29^ Additionally, Dünki et al. synthesized silicone elastomers functionalized with methylsulfonyl groups, achieving a high relative permittivity of 22.7.^30^ However, the *T*_g_ of these materials was found to be close to room temperature, which impacts elasticity and practical application. To address this, they incorporated butyl groups in varying ratios to effectively lower the *T*_g_. Moreover, Sheima et al. utilized methylsulfonyl-functionalized polysiloxanes to construct dielectric elastomer actuators (DEAs) capable of achieving a lateral actuation strain of 14% at 24.2 V μm^–1^, demonstrating their potential for actuator applications.^31^ Building on these advances, we present the synthesis and comprehensive evaluation of functionalized polysiloxanes using ethylsulfonyl instead of methylsulfonyl as lateral substituents. The ethylsulfonyl moiety was chosen due to the strong polarity of the sulfonyl group, which is anticipated to enhance the polymer’s dielectric permittivity, as has been observed with the methylsulfonyl group grafted to the polysiloxane. Moreover, the ethyl chain may disturb the dipolar interaction, increasing the mobility of the polymer. Additionally, the relatively short ethyl chain allows for a high concentration of polar sulfonyl groups, which increases the dielectric permittivity. We demonstrate that this small yet significant chemical modification substantially reduces the *T*_g_ of the polymers, as desired. In addition, the single-layer DEA constructed from this device showed an actuation strain of approximately 12% below 9 V μm^–1^, whereas the best-performing nitrile-functionalized and methyl sulfonyl-functionalized devices showed similar actuation strains when the electric field exceeded 20 V μm^–1^.^31,32^ Furthermore, by systematically tuning the butyl thioether content, we optimized the materials’ thermal, dielectric, and mechanical properties to enhance their performance for dielectric elastomer applications.

## 2 Results and Discussion

### 2.1 Synthesis of Polysiloxane Elastomers Modified with Ethylsulfonyl Groups

The synthetic strategy for polysiloxane elastomers modified with polar ethylsulfonyl groups is illustrated in Scheme 1. It starts with poly­(methylvinyl siloxane) (**PV**) (*M*_w_ = 85.3 kg mol^–1^, *M*_n_ = 58.3 kg mol^–1^, *D̵* = 1.46) containing about 12.24 wt % cycles, which was synthesized by anionic ring-opening polymerization of 1,3,5,7-tetravinyl-1,3,5,7-tetramethylcyclotetrasiloxane (Figure S1 and Table S1). The **PV** polymer, featuring lateral vinyl functional groups, was used to introduce polar side groups via a UV-assisted thiol–ene click reaction (Scheme 1).

**Scheme 1:**
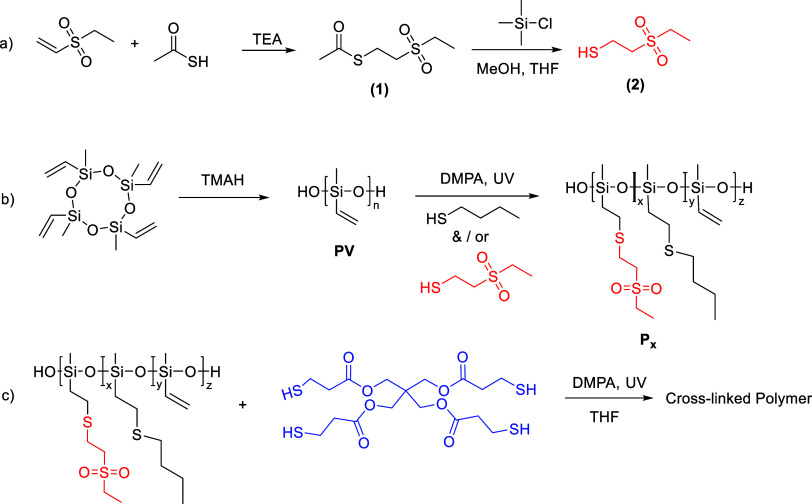
Synthesis of the Polar Thiol 2-(Ethylsulfonyl)­ethanethiol Used for Functionalization Employing a Thiolene Reaction of Ethyl Vinyl Sulfone with Thioacetic Acid Followed by the Deprotection of the Formed Thioester to the Thiol (a); Poly­(methylvinyl Siloxane) **PV** Synthesized by Ring-Opening Anionic Polymerization of 1,3,5,7-Tetravinyl-1,3,5,7-tetramethylcyclotetrasiloxane in the Presence of TMAH Followed by the UV-Induced Thiol–Ene Addition Which Grafts the Polar Groups to the Backbone to Give Polymer **P**_**x**_ with Different Contents of Sulfonyl Groups (b); And Cross-Linking of the Polymers by a UV-Induced Thiol–Ene Click Reaction, Using 2,2-Dimethoxy-2-phenylacetophenone (DMPA) as an Initiator and a Tetra-Functional Thiol Cross-Linker (c)

Two different groups, 2-(ethylsulfonyl)­ethylenethioether and butanethioether, were incorporated in different proportions to obtain polymers **P**_x_, where **x** represents the molar percentage of polar 2-(ethylsulfonyl)­ethanethiol in the mixture with butanethiol. ^1^H and ^13^C NMR spectra of the polar thiols used are presented (Figures S2 and S3). These polymers were characterized using ^1^H and ^13^C NMR spectroscopy, providing clear evidence for successful functionalization (Figures 1 and S4). Due to the great difference in polarity between **PV** and **P**_**x**_, the expected increase in the molar mass of **P**_**x**_ due to the chemical modification with thiols was not clearly observed in the GPC (Figure S1 and Table S1). This was because tetrahydrofuran (THF) was not a suitable solvent for conducting the GPC of the higher polarity polymers. For instance, the most polar polymer **P**_**100**_ exhibited a much lower hydrodynamic volume than the starting polymer **PV**. All polymers contained a small fraction of cycles/oligomers, which were difficult to remove by repetitive precipitation (Figure S1 and Table S1). During functionalization, a small fraction of the vinyl groups were left unreacted: 3 mol % in **P**_**25**_, 4 mol % in **P**_**50**_, 5 mol % in **P**_**75**_, and 8 mol % in **P**_**100**_. The residual vinyl groups enabled subsequent cross-linking of the polymers into elastomers using the tetrakis­(3-mercaptopropionate) cross-linker (Scheme 1).

**Figure 1:**
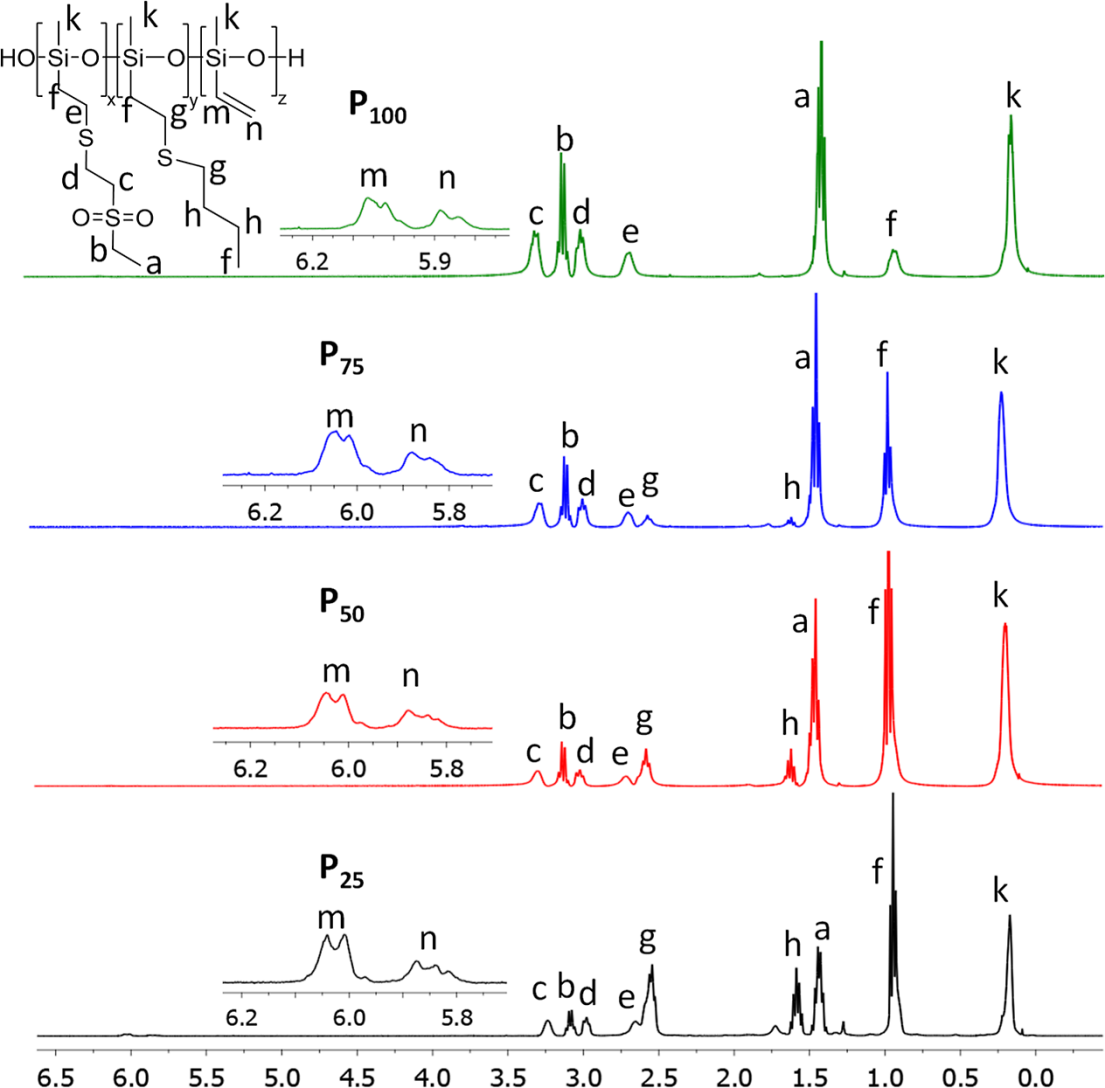
^1^H NMR spectra of the polymers **P**_**x**_ at room temperature in CDCl_3_ with signal assignment.

The optimal cross-linker concentrations required to cross-link the polymers into mechanically stable thin films were similar to those used by Sheima et al.^31^ and are summarized in Table 1. The concentrations were determined by using a 4-fold excess of cross-linker relative to the vinyl content of the polymer. The polymers were combined with the cross-linker, THF, and 2,2-dimethoxy-2-phenylacetophenone (DMPA) photoinitiator and mixed using a speed mixer for 5 min to form a homogeneous mixture, which was subsequently processed into thin films by doctor blading. The resulting films were then subjected to UV irradiation for 5 min to produce free-standing films designated as **M**_**25**_, **M**_**50**_, **M**_**75**_, and **M**_**100**_, according to the polymer **P**_**x**_ used for their synthesis. The cross-linker could react not only with the pendent vinyl groups but also form disulfide bridges, which were challenging to detect by infrared spectroscopy due to their low concentration. FTIR analysis showed the presence of different peaks characteristic for the polar group at 764 cm^–1^ for S–C, 1313 cm^–1^ for S = O, and 2933 cm^–1^ for C–H, and peaks characteristic for the polysiloxane backbone at 1283 cm^–1^ for Si–CH_3_, 1082 cm^–1^ for Si–O–Si, and 784 cm^–1^ for Si–C (Figure S5).

**Table 1:** Characteristics of Different Materials **M**_**x**_ Synthesized: The Content of Sulfonyl, Butyl, and Vinyl Groups of the Starting Polymers **P**_**x**_, Amount of Cross-Linker (CL) Used, the Thickness of the Films, the Glass-Transition Temperature (*T*_g_), Elastic Modulus at 10% Strain (*Y*_10%_), Storage Modulus (*E′*) at 0.05 Hz, and Dielectric Permittivity (ε′) at 10 kHz

M_**x**_	sulfonyl mol %^a^	butyl mol %^a^	vinyl mol %^a^	CL [mmol]	thickness [**μ**m]	*T*_g_ [°C]	*Y*_10%_ [kPa]	*E*′@0.05 Hz [kPa]	ε′@10 kHz
**M**_**25**_	26	71	3	5.6	105	–69.0	69.8 ± 0.7	170 ± 1.5	10.8 ± 0.4
**M**_**50**_	49	47	4	7.2	102	–52.6	174.0 ± 1.6	178 ± 0.6	13.2 ± 0.2
**M**_**75**_	70	25	5	8.0	121	–38.7	228.0 ± 6.3	262 ± 6.9	14.2 ± 0.1
**M**_**100**_	92		8	12.8	135	–27.9	184.0 ± 0.8	184 ± 16.5	16.2 ± 0.3

a Experimentally determined from ^1^H NMR spectra.

### 2.2 Thermal Characterization of **M**_**x**_

Thermogravimetric analysis (TGA) was conducted to assess the thermal stability of our materials (Figure 2a). It was carried out from 25 to 700 °C with a heating rate of 10 °C min^–1^ under a nitrogen atmosphere to prevent oxidative degradation. All polymers were stable up to 300 °C, at which point the first decomposition step was observed, resulting in a weight loss of 65 to 70%. The residue likely consists of silica.

**Figure 2:**
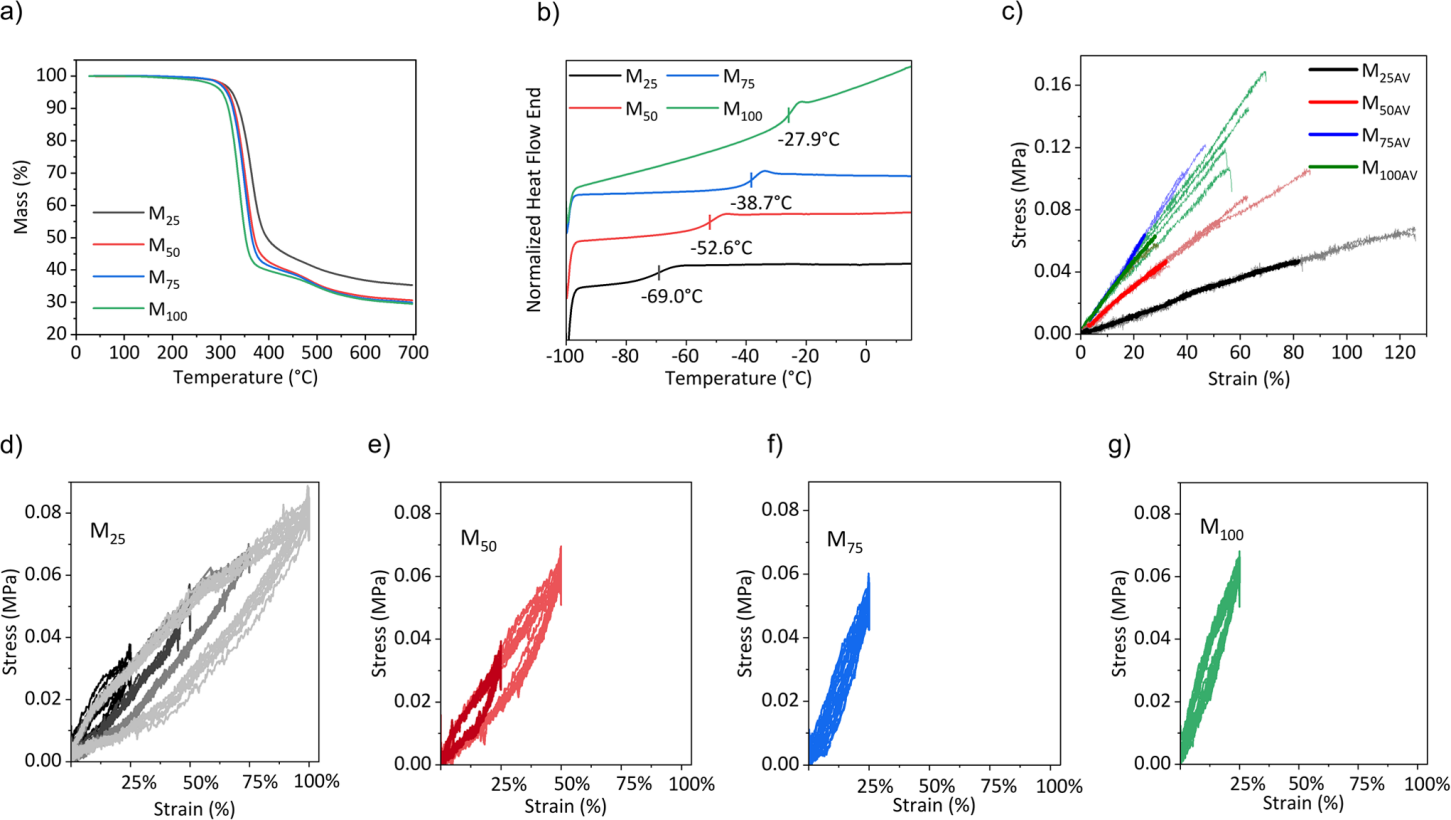
Thermogravimetric analysis of elastomers **M**_**x**_ at a heating rate of 10 °C min^–1^ under a nitrogen flow from 25 to 700 °C (a). DSC curves from −100 to 15 °C under a nitrogen flow (b). Stress–strain curves of materials **M**_**x**_ (c). Uniaxial cyclic tests over 10 cycles for **M**_**25**_ at 25%, 50%, 75%, and 100% strain (d); **M**_**50**_ at 25% and 50% strain (e); **M**_**75**_ at 25% strain (f); and **M**_**100**_ at 25% strain (g).

Differential scanning calorimetry (DSC) was carried out on all materials to determine their transition temperatures. A clear increase in *T*_g_ from −69 to −27.9 °C with increasing polarity of the materials was observed (Figure 2b). Still, the *T*_g_ of −27.9 °C for the most polar material was significantly lower than that measured for a recently reported polysiloxane elastomer modified with methylsulfonyl groups, which had a *T*_g_ = −13.6 °C.^31^ Thus, replacing the methyl group on the sulfonyl group with ethyl allowed us to reduce the *T*_g_ by about 14 K. A further reduction in *T*_g_ was possible by decreasing the content of sulfonyl groups on the polymer by using a mixture of 2-(ethylsulfonyl)­ethane-1-thiol and butanethiol in the postpolymerization modification reaction. As a result, materials containing 25, 50, and 75 mol % of the butyl sulfide group showed a decrease in *T*_g_ from −38.6 to −52.6 and −69 °C, respectively.

### 2.3 Mechanical Characterization of **M**_**x**_

The stress–strain behavior of the cross-linked materials was analyzed through tensile testing (Figure 2c), and Young’s modulus was determined from the slope of the stress–strain curve in the linear regime (0–10% strain) and summarized in Table 1. Material **M**_**25**_ demonstrated the highest average strain at break of 125%, followed by **M**_**50**_ at 86%, **M**_**100**_ at 69%, and **M**_**75**_ at 47%. As expected, the *Y*_10%_ increased with polarity from 70 kPa for **M**_**25**_, to 174 kPa for **M**_**50**_, and reached a maximum of 228 kPa for **M**_**75**_. However, material **M**_**100**_, despite its polarity, was softer than **M**_**75**_. This was probably because the *T*_g_ of this polymer was the highest and polymer mobility might not be sufficient, negatively affecting the cross-linking reaction. Therefore, the amount of cross-linker used to synthesize **M**_**100**_ was increased, leading to a softer network. Material **M**_**100**_ was similar to the one reported by Sheima et al., but the ethyl group in **M**_**100**_ was replaced by a methyl one. The material reported had a significantly lower strain at break (39%) and was over five times stiffer than **M**_**100**_, with a Young’s modulus of 1100 kPa at 10% strain. The enhanced softness of **M**_**100**_ compared with that reported by Sheima et al. might be due to the presence of ethyl groups on the polar group, which supposedly reduced the dipolar interaction and introduced more free volume, leading to increased chain mobility and a lower *T*_g_.

Successive tensile cycles were performed to assess any degradation in performance as the number of cycles progressed (Figure 2d–g). These tests repeatedly stretched the elastomers in a single direction to assess key mechanical properties such as hysteresis and strain recovery under cyclic loading. Each material underwent 10 cycles of stretching and relaxation, each lasting 10 s. **M**_**75**_ and **M**_**100**_ withstood a maximum elongation of 25%, while **M**_**50**_ withstood up to 50% elongation. Notably, **M**_**25**_ exhibited superior elongation, withstanding up to 100% strain. These findings were consistent with the tensile test results, which confirmed that **M**_**25**_ was the softest material and had the highest elongation at break. All materials exhibited some hysteresis loop, indicating energy loss, which was calculated for each material based on the area enclosed in the loading–unloading stress–strain loops. The energy loss at 20% strain increased with increasing polarity from 0.014 J cm^–3^ for **M**_**25**_, to 0.019 J cm^–3^ for **M**_**50**_, to 0.021 J cm^–3^ for **M**_**75**_, and reached a maximum value of 0.025 J cm^–3^ for **M**_**100**_. The energy dissipated by **M**_**25**_ increased with the strain level from 0.014 J cm^–3^ at 25%, to 0.038 J cm^–3^ at 50%, to 0.122 J cm^–3^ at 75%, and reached a maximum value of 0.203 J cm^–3^ at 100% strain. However, the elastomers returned to their original size after the stress was released.

Dynamic mechanical analysis (DMA) was performed to evaluate the storage modulus (*E*′), loss modulus (*E*″), and tan δ of the materials over the frequency range of 0.05 to 10 Hz at a strain of 2% at room temperature (Figure 3a). The storage modulus of all materials remained nearly constant between 0.05 and 1 Hz, indicating predominantly elastic behavior. However, at higher frequencies between 1 and 10 Hz, an increase in the *E*′ and mechanical losses were observed. At 0.05 Hz, the storage modulus of the materials followed a trend consistent with the results obtained from the tensile test, with **M**_**75**_ exhibiting the highest storage modulus and **M**_**25**_ the lowest. Additionally, the tan δ values provided insights into the mechanical performance of the materials. All materials exhibited very low mechanical losses at low frequencies with tanδ below 0.05. At 10 Hz, **M**_**25**_, **M**_**50**_, and **M**_**75**_ exhibited tan δ values below 0.2, indicating minimal mechanical losses and relatively good elasticity. In contrast, **M**_**100**_ demonstrated significant mechanical losses at higher frequencies, which are related to its high *T*_g_.

**Figure 3:**
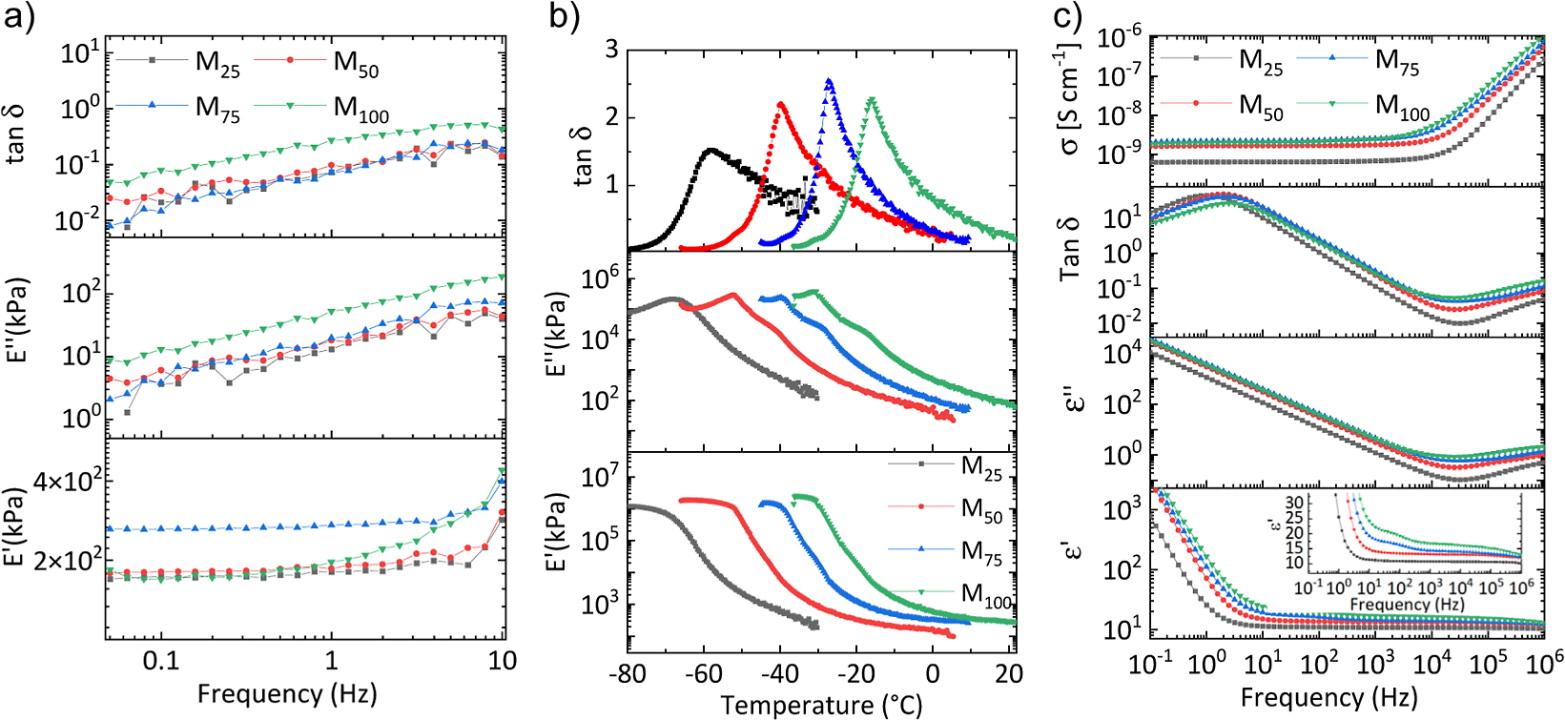
Dynamic mechanical analysis (DMA) frequency sweep from 0.05 to 10 Hz and 0.2% strain (a). DMA temperature sweeps at 0.05% strain at 1 Hz at different temperatures, illustrating the change from brittle to rubber (b). Broadband dielectric spectroscopy of **P**_**x**_ at room temperature with an AC frequency of 0.1 Hz up to 1 MHz with the conductivity (σ′), dielectric loss tangent (tan δ), and complex permittivity (ε″ and ε′) (c).

Temperature-dependent DMA provides valuable information about the changes in mechanical properties with temperature and *T*_g_. The samples were subjected to 0.05% strain at 1 Hz while varying the temperature (Figure 3b). Below *T*_g_, the storage modulus of all materials was above 1 GPa, and it dropped several orders of magnitude after passing through *T*_g_. The peak in tan δ curve corresponds to the *T*_g_. Among the samples, **M**_**25**_ demonstrated the highest thermal stability at lower temperatures, as observed from the tan δ curve. The *T*_g_ values were as follows: −16.8 °C for **M**_**100**_, −27.2 °C for **M**_**75**_, −39.9 °C for **M**_**50**_, and −58.5 °C for **M**_**25**_. Above the *T*_g_, the storage modulus was higher than the loss modulus, which supported the fact that our materials were chemically cross-linked. For all materials, the tan δ exhibited a non-Gaussian shape, indicating the presence of another process occurring near the *T*_g_. This process was caused by the adsorbed water, which was difficult to remove due to the polarity of the material. The presence of water was further confirmed by the temperature-dependent impedance spectroscopy measurements (Figure 4c). Such phenomena are well documented in the literature.^33^

**Figure 4:**
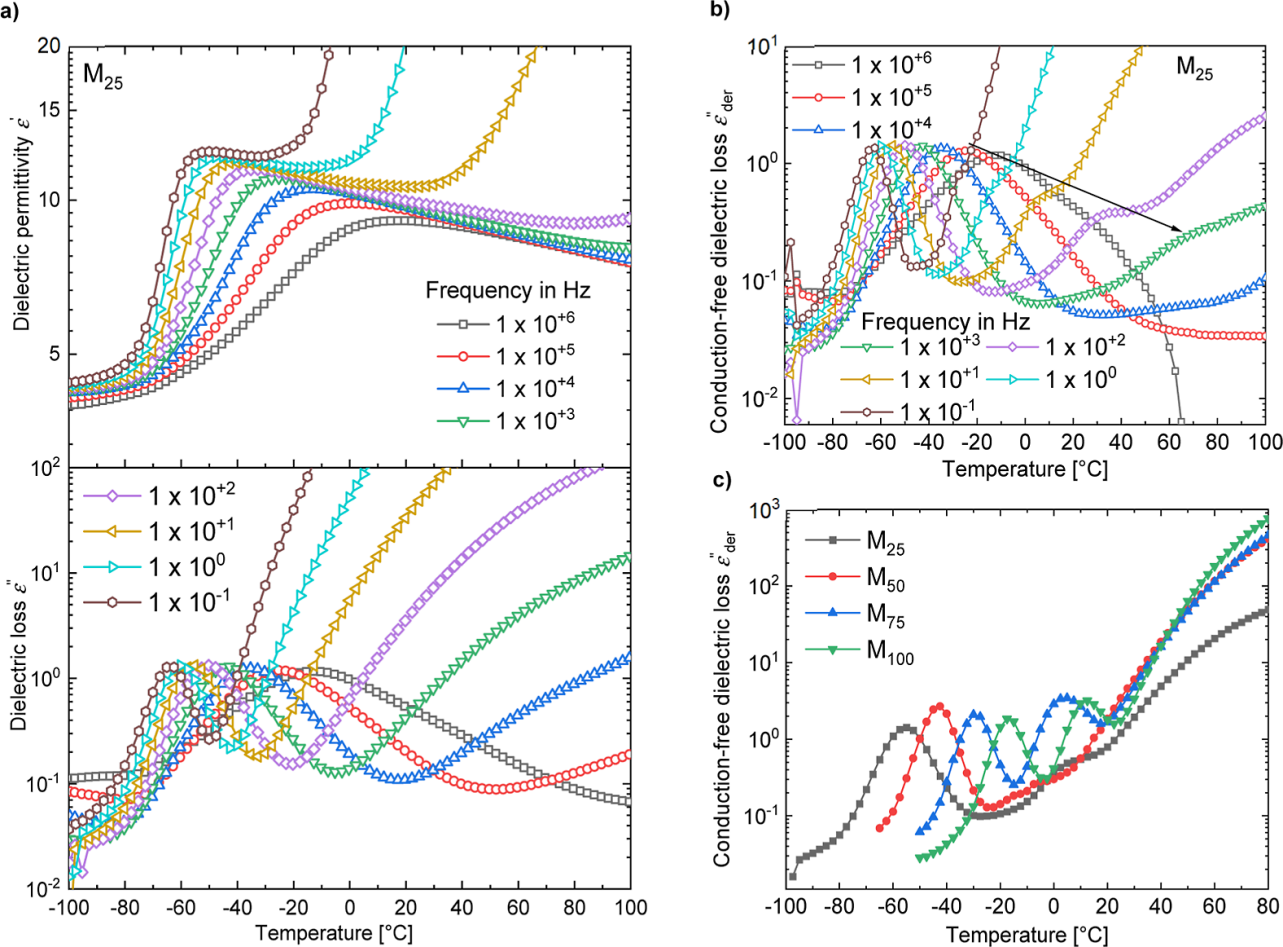
Real and imaginary parts of the permittivity of **M**_**25**_ are plotted as a function of temperature at selected frequencies (a). Isochronal representation of the conduction-free dielectric loss (ε_der_^″^) calculated for the **M**_**25**_ sample at selected frequencies (b). ε_der_^″^ curves of all materials are displayed at 10 Hz, and 2nd *T*_g_ in all the samples are observed (c).

### 2.4 Impedance Spectroscopy Characterization of **M**_**x**_

The dielectric properties of the polymers with varying ratios of polar groups were evaluated using dielectric impedance spectroscopy. The measured parameters, including dielectric permittivity (ε′), dielectric loss (ε″), dielectric loss tangent (tan δ), and conductivity (σ′), were presented as functions of frequency (Figure 3c). All materials were electrically insulating with a conductivity below 2.6 × 10^–9^ S cm^–1^ at frequencies below 1 kHz. Above 10 kHz, a steep increase in conductivity and a corresponding decrease in impedance were observed.^34^ This occurred because the electric field alternated so rapidly that dipoles in the material could not fully align with the field anymore before it changed direction.^35^ As a result, energy dissipation through polarization mechanisms was reduced. Instead, the material behaved more like a conductor, allowing charges to move more freely in response to the rapidly oscillating field. At low frequencies, the permittivity and the imaginary part of the permittivity ε″ were inversely proportional to the frequency (with a slope of −1 in the double logarithmic plot), a behavior attributed to electrode polarization. This phenomenon arised from the accumulation of ionic residues at the electrode interfaces, resulting in interfacial polarization, which overlapped with the orientation polarization of the polymer.^36,37^ **M**_**25**_ exhibited the lowest conductivity down to 6.5 × 10^–10^ S cm^–1^ and the lowest dielectric loss over a broad frequency range. As expected, the dielectric permittivity increased with the content of polar ethylsulfonyl groups. Consequently, **M**_**100**_ exhibited the highest permittivity at 10 kHz of 16.2 ± 0.35, followed by **M**_**75**_ of 14.2 ± 0.16, **M**_**50**_ of 13.2 ± 0.13, and **M**_**25**_, which had the lowest permittivity of 10.8 ± 0.28.

Temperature-dependent dielectric measurements revealed how the dielectric properties varied with temperature (from −100 to 100 °C for **M**_**25**_, −65 to 100 °C for **M**_**50**_, −50 to 100 °C for **M**_**75**_ and **M**_**100**_) and frequency (from 0.1 Hz to 1 MHz) (Figures S6–S9). To better visualize the changes, the real and imaginary parts of the permittivity of **M**_**25**_ were plotted as a function of temperature at selected frequencies (Figure 4a). At −80 °C, the sample was below its *T*_g_, where the dipoles were frozen. Hence, the permittivity was low. As the polymer passed through its *T*_g_, the dipoles became mobile, which was reflected in an increase in the permittivity due to orientation polarization. In the loss plot, corresponding relaxation peaks were observed, which were shifted to higher frequencies with increasing temperature. Above the glass transition, at low frequencies and high temperatures, we observed a significant increase in both permittivity and losses, attributed to the enhanced ionic conductivity, which led to electrode polarization.

In Figure 3c, we could observe that samples M75 and M100 exhibited a step in permittivity around 100 Hz, indicating an additional relaxation. However, we could not observe corresponding loss peaks due to the increased conductivity at these frequencies. Using derivative techniques, it was possible to remove the contribution from d.c. conductivity.^38,39^ eq 1 shows the frequency derivative of permittivity, which is proportional to the d.c. conduction-free dielectric loss (ε_der_^″^). Figure 4b shows the ε_der_^″^ as a function of temperature at selected frequencies. In addition to the glass-transition relaxation seen in the dielectric loss curves at low temperatures (Figure 4a), we clearly saw the presence of an additional relaxation at higher temperatures. A similar observation has been made in polysiloxane copolymers modified with a similar butyl group and a cyclic sulfonyl, as reported in a previous study.^22,34^ In both these cases, fitting the glass transition and the additional relaxation loss peaks by the Havriliak and Negami (HN) equation, which is used for analyzing relaxations in polymers, yielded a nonlinear VFT fit.^34^ This indicated two glass transitions in the copolymer system similar to the one under investigation. The nonlinear frequency dependence of the second relaxation could be deduced from the 3D ε_der_^″^ plot of the **M**_**25**_ sample as a function of temperature and frequencies (Figure S10). At first glance, though two *T*_g_ values suggest that the **M**_**25**_ random copolymer is phase segregated (Figure 4c),^40^ we could also observe a second *T*_g_ in **M**_**100**_, which was a homopolymer. Comparison of the ε_der_^″^ curves of different samples at a frequency of 10 Hz indicated the presence of the second *T*_g_ in all the samples (Figure 4c).

1 $$\varepsilon_{der}^{\prime \prime }=-\frac{\pi }{2}\frac{\partial \varepsilon^{\prime }\left(\omega \right)}{\partial \ln\omega }$$

In agreement with the DSC measurements, the primary *T*_g_ peaks shifted to higher temperatures with increased polarity. Based on the HN-fit parameters and other detailed analyses,^34^ the second *T*_g_ was caused by the interaction of water molecules with the dipoles. Previously, a similar additional *T*_g_ was detected in protein–water mixtures.^33^ It was explained as a result of microphase segregation, where regions were plasticized to varying extents by the water molecules. The extent of this interaction depended on the sample’s polarity, as indicated by the shift in the second *T*_g_ with respect to the primary relaxation. While in the less polar **M**_**25**_ and **M**_**50**_ samples, the second *T*_g_ appeared farther from the primary *T*_g_ peak as a shoulder, it was observed closer and as a strong peak in the more polar samples. This was in accordance with the temperature-dependent *E*″ curves measured by DMA (Figure 3b), where we observed a weak shoulder for a **M**_**50**_ sample around −40 °C, and a stronger shoulder for the polar **M**_**75**_ and **M**_**100**_ samples at higher temperatures.

### 2.5 Single-Layer Actuator Characterization

**M**_**100**_ and **M**_**75**_ were selected for constructing single-layer actuators due to their favorable properties, which include high elasticity demonstrated through dynamic mechanical analysis, good strain at break observed in tensile testing, and the highest dielectric permittivities among the samples. Single-layer actuators made from a 107 μm thick film of **M**_**75**_ and a 110 μm thick film of **M**_**100**_ were subjected to stepwise voltage increases until dielectric breakdown was reached (Figure 5a). At an electric field of 8.2 V μm^–1^, **M**_**100**_ exhibited a lateral actuation strain of 11.8%, while **M**_**75**_ showed at an electric field of 8.4 V μm^–1^ a maximum actuation strain of 6.0%, nearly half than that of **M**_**100**_. Since the two actuators have similar thicknesses, the difference could be attributed to **M**_**100**_’s higher dielectric permittivity and lower elastic modulus. The dielectric strength of **M**_**100**_ was measured using two 1 mm^2^ rigid metal electrodes embedded in an epoxy resin. The Weibull probability plot (Figure 5b) presents the statistical distribution of the dielectric strength, providing valuable information on the reliability of this material as a dielectric. It was created using 10 measurements and Origin software. The slope parameter β = 6.2 indicated wear-out failure, while the scale parameter of 19.7 represented the breakdown field at which 63.2% of samples failed and the maximum operation electric field in the actuator.

**Figure 5:**
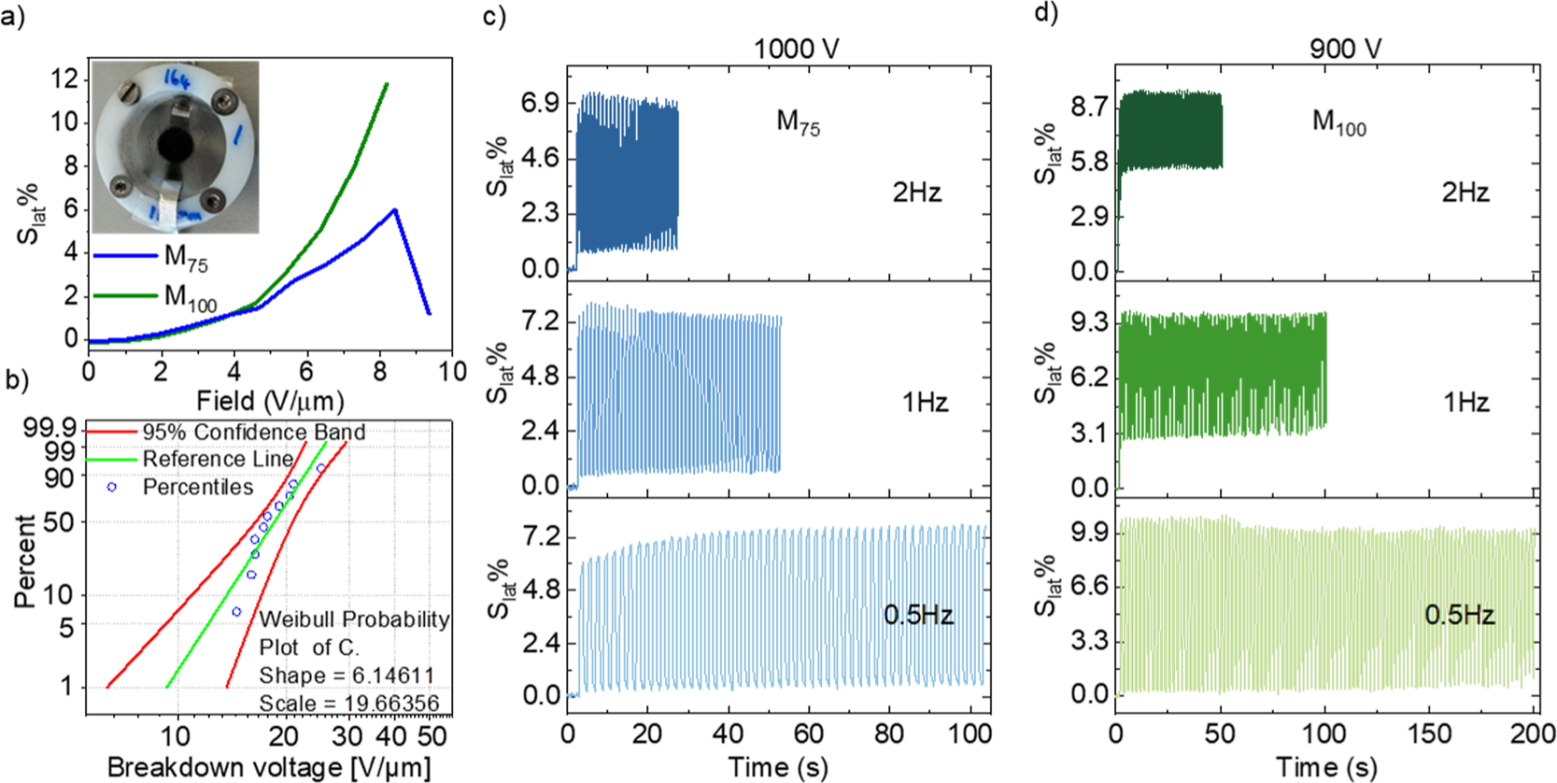
Lateral actuation strain at different electric fields up to the dielectric failure of **M**_**75**_ and **M**_**100**_ (a). Weibull probability plot of **M**_**100**_ (b). **M**_**75**_ actuator was tested at 1000 V with frequencies of 0.5, 1, and 2 Hz (c). **M**_**100**_ actuator was tested at 900 V with frequencies of 0.5, 1, and 2 Hz (d).

These results aligned with the behavior of polar-modified polysiloxanes. Increasing polar group content enhanced dielectric permittivity, boosting electrostatic pressure and significantly improving actuation performance—up to ∼12% strain in our materials, compared to <0.5% in unmodified Elastosil.^34^ However, this improvement was accompanied by a reduction in dielectric breakdown strength (less than 20 V μm^–1^ compared to about 90 V μm^–1^ for Elastosil) and higher dielectric losses due to increased polarity.^41^ However, through effective cross-linking strategies, careful voltage application, and operation at lower frequencies, we achieved large actuation strains with stable performance over repeated cycles without breakdown.

Both **M**_**75**_ and **M**_**100**_ actuators underwent cyclic actuation testing at different frequencies under an electric field of 9.4 V μm^–1^ (1000 V) and 8.2 V μm^–1^ (900 V), respectively (Figure 5c,d). At a frequency of 0.5 Hz, both actuators demonstrated relatively stable performance for 50 cycles, with actuation strains of 7.6% for **M**_**75**_ and 10.8% for **M**_**100**_, respectively, exhibiting no hysteresis between cycles. However, the two materials **M**_**75**_ and **M**_**100**_ behaved slightly differently in actuators for which the frequency of operation was 1 and 2 Hz. The actuator constructed from **M**_**75**_ exhibited a stable and reversible actuation at both frequencies. The actuator had sufficient time to return to the initial shape. This suggested that the operation time was sufficient to allow the dipoles in **M**_**75**_ to return to their initial random state. Material **M**_**100**_ exhibited a maximum actuation of approximately 9.3% at 1 Hz; however, when operated at this frequency, the actuator did not return to its initial state but rather remained slightly actuated due to delayed mechanical recovery resulting from viscoelastic relaxation. Although the amplitude of actuation was only 6.9% at 1 Hz, after a few cycles, the actuation was stable, and no hysteresis between the cycles could be observed. For comparison, a 100 μm thick actuator made from a polysiloxane elastomer modified with methylsulfonyl groups, as reported by Sheima et al., showed an actuation strain of less than 2% under an electric field of 6 V μm^–1^ (600 V) at 1 Hz and suffered irreversible damage at 10 V μm^–1^ (1000 V). The superior performance of **M**_**100**_ could be attributed to the ethyl substituent on the sulfonyl group, which lowered the *T*_g_ and thus enhanced backbone flexibility, thereby improving actuation under similar electric fields.

When the actuator was operated at 2 Hz, a significantly stronger decrease in actuation amplitude to 4.2% was measured. These results suggested that the mechanical losses of **M**_**100**_ were responsible for actuator behavior when operated at 1 and 2 Hz. The frequency-dependent variation in actuation performance was not unexpected, given the increased losses observed at higher frequencies for material **M**_**100**_ (Figure 3a).

### 2.6 Stack Actuator Characterization

Due to the large and reversible actuation at 0.5 Hz of **M**_**100**_, a stack actuator was also constructed by manually stacking five dielectric elastomer films and six electrode layers in an alternating manner (Figure 6a). The overall thickness of the stack was 1150 μm. The elastic electrode material was synthesized, as previously described,^42^ through the anionic ring-opening polymerization of a cyclic silicone monomer containing cyanopropyl groups and a polar bicyclic cross-linker, using tetramethylammonium hydroxide (TMAH) as the initiator. Polymerization and cross-linking occurred simultaneously, forming an elastomer network in a single step. Performance testing involved applying a voltage and measuring the displacement with a laser, offering precise insights into the stack actuator’s response. When a voltage was applied, the electrostatic pressure generated by the electrodes compressed the elastomer layers in the cross-plane direction, reducing the stack’s overall thickness. Upon voltage removal, the actuator returned to its original thickness due to its elastic recovery. The actuator was actuated at 1200 V across various frequencies: 1, 2, 5, and 10 Hz (Figure 6b). As the frequency increased, the strain decreased from ∼34 μm at 1 Hz, to ∼28 μm at 2 Hz, to ∼22 μm at 5 Hz, and to ∼17 μm at 10 Hz. Despite this, the device exhibited stable actuation across all frequencies. When the applied voltage was increased to 1400 and 1600 V at 1 Hz (Figure 6c,d), the actuator demonstrated thickness changes of 40 and 52 μm, respectively. At 1400 V, stable actuation was observed over 92 cycles. However, when the voltage was increased to 1600 V, the stack suffered several dielectric breakdowns, reflected in a decay in actuation after 24 cycles. However, the actuator self-healed and continued to operate stably for over 88 cycles. The thickness change of 52 μm at 1600 V corresponded to an in-thickness actuation strain of 4.5% under an electric field of 14.5 V μm^–1^.

**Figure 6:**
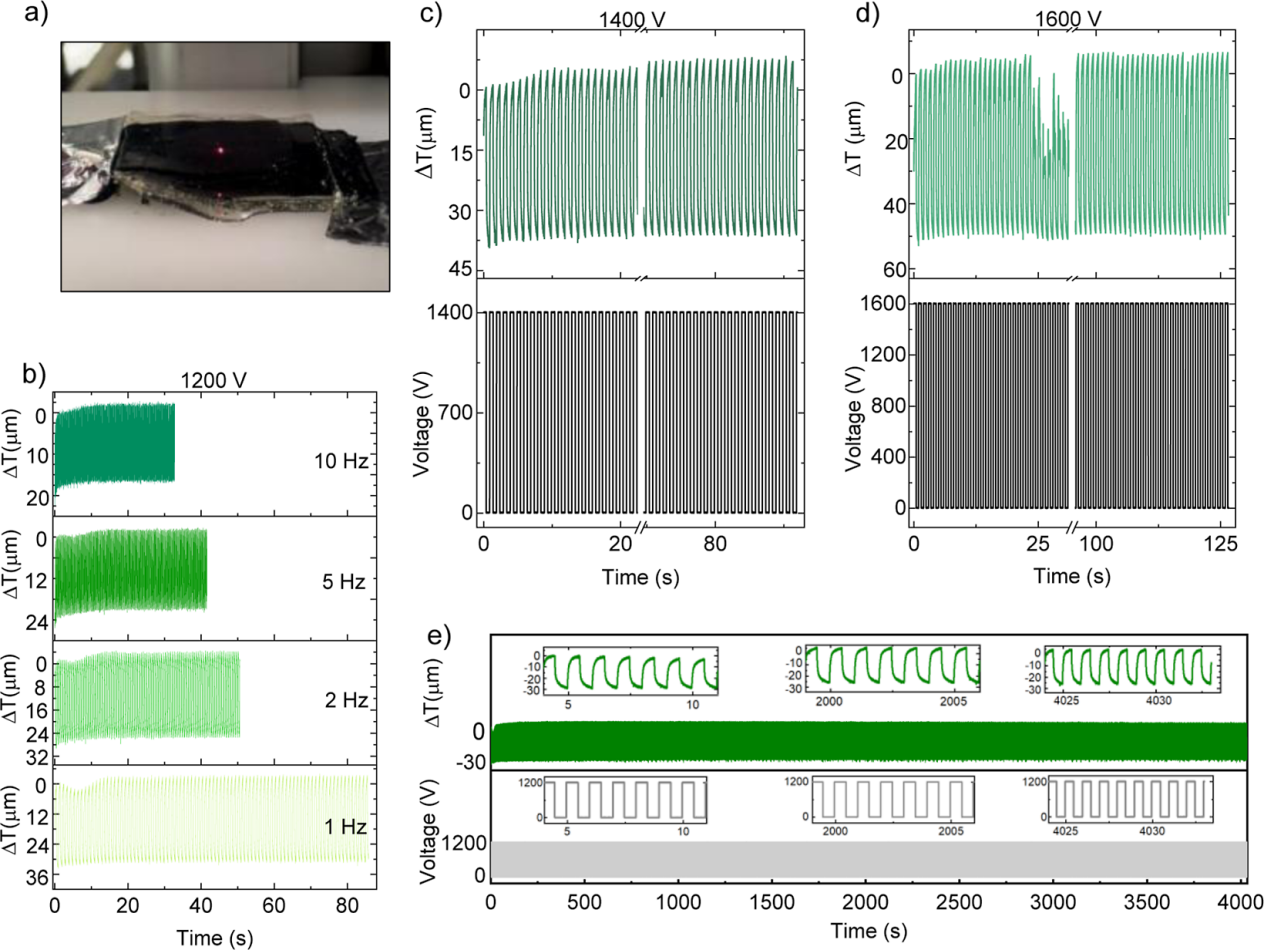
(a) A photo of the stack actuator. (b) Stack actuator constructed from **M**_**100**_ was tested at 1200 V with frequencies of 1, 2, 5, and 10 Hz for 86, 100, 205, and 320 cycles, respectively. (c) Stack actuator operated at 1 Hz and 1400 V for 92 cycles and (d) at 1600 V for 126 cycles. (e) The actuators suffered several dielectric breakdowns when operated at 1600 V. However, they could self-heal and recover the initial actuation. The stack was tested at 1200 V with a frequency of 1 Hz and displayed stable actuation of more than 4000.

After all these tests, the stack was tested at 1200 V for over 4000 cycles and showed a stable actuation (Figure 6e), which demonstrated that our material was quite robust and could tolerate some dielectric breakdowns without losing performance. In future work, the generated force should be enhanced by increasing the number of active layers to achieve larger stacks, reducing the thickness of the dielectric film, and increasing the dielectric breakdown following further optimization of material processing.

## 3 Experimental Section

### 3.1 Materials

Unless otherwise specified, all chemicals were used as purchased. Trimethylamine, hydrochloric acid, methanol (MeOH), dichloromethane, THF, magnesium sulfate (MgSO_4_), sodium bicarbonate (NaHCO_3_) and 2,2-dimethoxy-2-phenylacetophenone (DMPA) were procured from VWR. Thioacetic acid, tetramethylammonium hydroxide (TMAH), butanethiol, pentaerythritol tetrakis­(3-mercaptopropionate), and benzene were purchased from Sigma-Aldrich. Additionally, ethyl vinyl sulfone, trimethylsilyl chloride (TMSCl), and 1,3,5,7-tetravinyl-1,3,5,7-tetramethylcyclotetrasiloxane were supplied by ABCR.

### 3.2 Syntheses

#### 3.2.1 Synthesis of *S*-(2-(Ethylsulfonyl)­ethyl) Ethanethiolate (**1**)

In a two-necked round-bottom flask, ethyl vinyl sulfone (15.65 mL, 18 g, 149.79 mmol, 1 equiv) and thioacetic acid (12.79 mL, 13.68 g, 179.75 mmol, 1.2 equiv) were dissolved in THF (200 mL). Trimethylamine (25.05 mL, 18.19 g, 179.75 mmol, 1.2 equiv) was added dropwise at room temperature, and the reaction mixture was allowed to vigorously stir for 4 h. Subsequently, hydrochloric acid (5.46 mL, 2 mol/L, 179.75 mmol, 1.2 equiv) was added to the reaction and the mixture was allowed to stir for additional half an hour. After removing the solvent, the slurry was dissolved in CH_2_Cl_2_ (200 mL) and washed with water three times. The collected organic layers were dried over MgSO_4_, filtered, and concentrated using a rotary evaporator. The product was obtained as a brown solid with a yield of 92.2% (27.1 g, 138.07 mmol). ^1^H NMR (400 MHz, CDCl_3_, δ): 3.33–3.16 (m, 4H, S–CH_2_–CH_2_–SO_2_), 3.07 (q, *J* = 7.5 Hz, 2H, SO_2_–CH_2_–CH_3_), 2.37 (s, 3H, CH_3_–CO), 1.44 (t, *J* = 7.5 Hz, 3H, SO_2_–CH_2_–CH_3_)·^13^C NMR (100 MHz, CDCl_3_, δ): 194.99 (−CO–S), 51.56 (S–CH_2_–CH_2_–SO_2_), 47.52 (SO_2_–CH_2_–CH_3_), 30.55 (CH_3_–CO), 21.97 (S–CH_2_–CH_2_–SO_2_), 6.64 (SO_2_–CH_2_–CH_3_).

#### 3.2.2 Synthesis of 2-(Ethylsulfonyl)­ethane-1-thiol (**2**)

In a two-necked round-bottom flask equipped with a condenser, **1** (26 g, 132.46 mmol, 1 equiv) was dissolved in a mixture of THF (100 mL) and MeOH (75 mL) at 45 °C. Then, TMSCl (1.2 mL, 1.44 g, 13.25 mmol, 0.1 equiv) was added dropwise, and the reaction mixture was stirred at 50 °C for 24 h. After removing the solvent at the rotary evaporator, the slurry was dissolved in CH_2_Cl_2_ and washed with saturated NaHCO_3_ solution. The aqueous layer was vigorously extracted with CH_2_Cl_2_ two times, and the collected organic layers were dried over MgSO_4_, filtered, and the solvent removed under reduced pressure. The residue was distilled at 2.1 mbar and 130 °C. The liquid product was obtained with a yield of 79.9% (16.32 g, 105.88 mmol). ^1^H NMR (400 MHz, CDCl_3_, δ): 3.24 (q, *J* = 8.9, 6.4 Hz, 2H, SO_2_–CH_2_–CH_3_), 3.11–2.93 (m, 4H, S–CH_2_–CH_2_–SO_2_), 1.79 (t, *J* = 8.5 Hz, 1H, HS), 1.42 (t, *J* = 7.5 Hz, 3H, SO_2_–CH_2_–CH_3_). ^13^C NMR (100 MHz, CDCl_3_, δ): 55.33 (S–CH_2_–CH_2_–SO_2_), 48.10 (SO_2_–CH_2_–CH_3_), 16.81 (S–CH_2_–CH_2_–SO_2_), 6.60 (SO_2_–CH_2_–CH_3_).

#### 3.2.3 General Method for Functionalizing Polymethylvinylsiloxane (**PV**)

Polymer **PV** was dissolved in THF, followed by adding butanethiol and/or 2-(ethylsulfonyl)­ethane-1-thiol under an argon atmosphere. DMPA (93.04 mg, 0.384 mmol, 0.01 equiv) was then added to the reaction mixture. After three freeze–thaw–pump cycles, the mixture was irradiated with UV light for 5 min. Subsequently, the solvent was removed under reduced pressure, and the resulting mixture was precipitated into methanol. The precipitate was redissolved in a minimal volume of THF and reprecipitated in methanol. The purified polymers **P**_**x**_ were obtained after drying under vacuum (Scheme 1).

Polymer **P**_**25**_: ^1^H NMR (400 MHz, CDCl_3_, δ): 6.08–5.75 (m, *J* = 67.5, 14.3 Hz, 3H, Si– CH = CH_2_), 3.23 (q, *J* = 8.9, 6.4 Hz, 2H, S–CH_2_–CH_2_–SO_2_), 3.07 (q, *J* = 7.4 Hz, 2H, SO_2_–CH_2_–CH_3_), 2.96 (t, *J* = 8.5 Hz, 2H, S–CH_2_–CH_2_–SO_2_), 2.65 (m, 2H, Si–CH_2_–CH_2_–S), 2.61–2.43­(m, 4H, Si–CH_2_–CH_2_–S–CH_2_), 1.63–1.50 (m, 4H, S–CH_2_–CH_2_–CH_2_–CH_3_), 1.42 (t, *J* = 7.5 Hz, 3H, SO_2_–CH_2_–CH_3_), 0.92 (m, 7H, Si–CH_2_–CH_2_–S and S–CH_2_–CH_2_–CH_2_–CH_3_), 0.16 (s, 3H, CH_3_–Si). ^13^C NMR (100 MHz, CDCl_3_, δ): 52.03 (S–CH_2_–CH_2_–SO_2_), 47.86 (SO_2_–CH_2_–CH_3_), 31.70 (S–CH_2_–CH_2_–CH_2_–CH_3_), 26.91 (Si–CH_2_–CH_2_–S–CH_2_–CH_2_–SO_2_), 26.40 (Si–CH_2_–CH_2_–S–CH_2_–CH_2_–CH_2_–CH_3_), 23.72 (S–CH_2_–CH_2_–SO_2_ and S–CH_2_–CH_2_–CH_2_–CH_3_), 22.05 (S–CH_2_–CH_2_–CH_2_–CH_3_), 18.39 (Si–CH_2_–CH_2_–S–CH_2_–CH_2_–SO_2_), 18.09 (Si–CH_2_–CH_2_–S–CH_2_–CH_2_–CH_2_–CH_3_), 13.78 (S–CH_2_–CH_2_–CH_2_–CH_3_), 6.61 (SO_2_–CH_2_–CH_3_), 0.01 (CH_3_–Si). The GPC revealed that *M*_n_ = 57.2 kg/mol, *M*_w_ = 88.1 kg/mol, *D̵* = 1.54, oligomers and cycles wt % was 3.2.

Polymer **P**_**50**_: ^1^H NMR (400 MHz, CDCl_3_, δ): 6.10–5.73 (m, *J* = 67.5, 14.3 Hz, 3H, Si– CH = CH_2_), 3.23 (q, *J* = 8.9, 6.4 Hz, 2H, S–CH_2_–CH_2_–SO_2_), 3.07 (q, *J* = 7.4 Hz, 2H, SO_2_–CH_2_–CH_3_), 2.96 (t, *J* = 8.5 Hz, 2H, S–CH_2_–CH_2_–SO_2_), 2.65 (m, 2H, Si–CH_2_–CH_2_–S), 2.61–2.43­(m, 4H, Si–CH_2_–CH_2_–S–CH_2_), 1.64–1.47 (m, 4H, S–CH_2_–CH_2_–CH_2_–CH_3_), 1.41 (t, *J* = 7.5 Hz, 3H, SO_2_–CH_2_–CH_3_), 0.93 (m, 7H, Si–CH_2_–CH_2_–S and S–CH_2_–CH_2_–CH_2_–CH_3_), 0.17 (s, 3H, CH_3_–Si). ^13^C NMR (100 MHz, CDCl_3_, δ): 51.98 (S–CH_2_–CH_2_–SO_2_), 47.76 (SO_2_–CH_2_–CH_3_), 31.69 (S–CH_2_–CH_2_–CH_2_–CH_3_), 26.93 (Si–CH_2_–CH_2_–S–CH_2_–CH_2_–SO_2_), 26.37 (Si–CH_2_–CH_2_–S–CH_2_–CH_2_–CH_2_–CH_3_), 23.75 (S–CH_2_–CH_2_–SO_2_ and S–CH_2_–CH_2_–CH_2_–CH_3_), 22.11 (S–CH_2_–CH_2_–CH_2_–CH_3_), 18.36 (Si–CH_2_–CH_2_–S–CH_2_–CH_2_–SO_2_), 18.06 (Si–CH_2_–CH_2_–S–CH_2_–CH_2_–CH_2_–CH_3_), 13.79 (S–CH_2_–CH_2_–CH_2_–CH_3_), 6.60 (SO_2_–CH_2_–CH_3_), 0.04 (CH_3_–Si). The GPC revealed that *M*_n_ = 43.9 kg mol^–1^, *M*_w_ = 75.3 kg mol^–1^, *D̵* = 1.71, oligomers and cycles wt % was 5.2.

Polymer **P**_**75**_: ^1^H NMR (400 MHz, CDCl_3_, δ): 6.12–5.76 (m, *J* = 67.5, 14.3 Hz, 3H, Si–CH = CH_2_), 3.23 (q, *J* = 8.9, 6.4 Hz, 2H, S–CH_2_–CH_2_–SO_2_), 3.07 (q, *J* = 7.4 Hz, 2H, SO_2_–CH_2_–CH_3_), 2.96 (t, *J* = 8.5 Hz, 2H, S–CH_2_–CH_2_–SO_2_), 2.66 (m, 2H, Si–CH_2_–CH_2_–S), 2.61–2.43­(m, 4H, Si–CH_2_–CH_2_–S–CH_2_),1.62–1.51 (m, 4H, S–CH_2_–CH_2_–CH_2_–CH_3_), 1.42 (t, *J* = 7.5 Hz, 3H, SO_2_–CH_2_–CH_3_), 0.93 (m, 7H, Si–CH_2_–CH_2_–S and S–CH_2_–CH_2_–CH_2_–CH_3_), 0.18 (s, 3H, CH_3_–Si). ^13^C NMR (100 MHz, CDCl_3_, δ): 51.96 (S–CH_2_–CH_2_–SO_2_), 47.74 (SO_2_–CH_2_–CH_3_), 31.69 (S–CH_2_–CH_2_–CH_2_–CH_3_), 26.87 (Si–CH_2_–CH_2_–S–CH_2_–CH_2_–SO_2_), 26.33 (Si–CH_2_–CH_2_–S–CH_2_–CH_2_–CH_2_–CH_3_), 23.75 (S–CH_2_–CH_2_–SO_2_ and S–CH_2_–CH_2_–CH_2_–CH_3_), 22.11 (S–CH_2_–CH_2_–CH_2_–CH_3_), 18.35 (Si–CH_2_–CH_2_–S–CH_2_–CH_2_–SO_2_), 18.08 (Si–CH_2_–CH_2_–S–CH_2_–CH_2_–CH_2_–CH_3_), 13.80 (S–CH_2_–CH_2_–CH_2_–CH_3_), 6.59 (SO_2_–CH_2_–CH_3_), 0.05 (CH_3_–Si). The GPC revealed that *M*_n_ = 45.1 kg mol^–1^, *M*_w_ = 75.0 kg mol^–1^, *D̵* = 1.66, oligomers and cycles wt % was 9.4.Polymer **P**_**100**_: ^1^H NMR (400 MHz, CDCl_3_, δ): 6.52–5.54 (m, *J* = 67.5, 14.3 Hz, 3H, Si– CH = CH_2_), 3.25 (q, *J* = 8.9, 6.4 Hz, 2H, S–CH_2_–CH_2_–SO_2_), 3.07 (q, *J* = 7.4 Hz, 2H, SO_2_–CH_2_–CH_3_), 2.96 (t, *J* = 8.5 Hz, 2H, S–CH_2_–CH_2_–SO_2_), 2.65 (m, 2H, Si–CH_2_–CH_2_–S), 1.40 (t, *J* = 7.4 Hz, 3H, SO_2_–CH_2_–CH_3_), 0.93 (t, 2H, Si–CH_2_–CH_2_–S), 0.19 (s, 3H, CH_3_–Si). ^13^C NMR (100 MHz, CDCl_3_, δ): 51.94 (S–CH_2_–CH_2_–SO_2_), 47.71 (SO_2_–CH_2_–CH_3_), 26.84 (Si–CH_2_–CH_2_–S–CH_2_–CH_2_–SO_2_), 23.70 (S–CH_2_–CH_2_–SO_2_), 18.07 (Si–CH_2_–CH_2_–S–CH_2_–CH_2_–SO_2_), 6.59 (SO_2_–CH_2_–CH_3_), 0.12 (CH_3_–Si). The GPC revealed that *M*_n_ = 36.0 kg mol^–1^, *M*_w_ = 61.9 kg mol^–1^, *D̵* = 1.71, oligomers and cycles wt % was 9.8.

### 3.3 Cross-Linker Solution Preparation

Pentaerythritol tetrakis­(3-mercaptopropionate) (0.51 g, 400.00 μL) was weighed into a vial and dissolved in 2000 μL of THF to create a 1:5 (v/v) solution of cross-linker in THF. Each elastomeric film was prepared using a freshly made cross-linker solution stored in a brown bottle.

### 3.4 Thin Film Formation

A homogeneous solution was prepared by mixing polymer **P**_**x**_ (1 g), the cross-linker (amounts used are listed in Table 1), and the DMPA initiator (4.5 mg) in a minimal volume of THF (1 mL) using a speed mixer at 3000 rpm for 5 min. The resulting mixture was then blade-cast onto a sacrificial layer of poly­(vinyl alcohol) (PVA) that had been prepared on a glass substrate. After the solvent evaporated, the polymer films were exposed to UV irradiation for 5 min to induce cross-linking, forming elastic films. The elastomer film on PVA was detached from the glass substrate and placed in a preheated water bath at 50 °C to dissolve the PVA. The washing was repeated three times. The cross-linked films were dried in a vacuum oven at 60 °C for 24 h before use.

#### 3.4.1 Characterization

^1^H and ^13^C spectra were recorded using a Bruker AV-III 400 spectrometer (Bruker BioSpin AG, Switzerland) equipped with a 5 mm CryoProbe Prodigy. The measurements were conducted at 400.2 MHz for ^1^H and 100.6 MHz for ^13^C. All experiments were performed at 298 K, employing standard Bruker pulse programs and parameter sets. Chemical shifts (δ) in NMR were referenced to residual solvent signals.

Tensile and cyclic uniaxial stress tests were performed on a Zwick Z010 testing machine at a crosshead speed of 50 mm min^–1^. Test specimens were prepared with a gauge width of 20 mm and a gauge length of 18.00 mm using a bone-shaped die-cutting technique. The strain was measured with a traversing sensor. The stress–strain curves were averaged from five samples for each material using Origin software. The tensile modulus was calculated from the slope of the stress–strain curves by applying a linear fit to the data points within the 0–10% strain range. The cyclic test of the materials was conducted for 10 cycles, with the samples held for 10 s in both the stretched and relaxed positions until the material failed.

Dielectric permittivity measurements were performed using a Novocontrol Alpha dielectric analyzer, with the sample temperature controlled by a Novocontrol Quatro cryosystem in a dry nitrogen atmosphere. Uniform films were placed between two 10 mm diameter stainless-steel electrodes. The dielectric permittivity of the films was measured over the frequency range from 10^–1^ to 10^6^ Hz.

DMA measurements were conducted using an RSA 3 DMA from TA Instruments. Uniform films, cut into 6 mm wide and 10 mm long stripes, were subjected to a 2.5 g dynamic load at 2% strain, with the frequency scanned between 0.05 and 10 Hz at 298 K. For temperature-dependent DMA tests, the same dynamic load and frequency range were applied to the samples at 0.05% strain at 1 Hz.

DSC experiments were carried out on a PerkinElmer Pyris Diamond. Approximately 10 mg of the sample was carefully weighed and placed in aluminum crucibles with perforated lids. Each analysis consisted of two heating cycles and one cooling cycle, with a heating and cooling rate of 10 °C min^–1^, spanning the temperature range from −60 to 100 °C, all performed under a nitrogen atmosphere. The second cooling cycle was used to determine the *T*_g_.

TGA measurements were conducted using a PerkinElmer TGA7, with a heating rate of 10 °C min^–1^ under a nitrogen flow from 25 to 700 °C.

GPC measurements in THF were conducted using an Agilent 1260 Infinity system equipped with two tandem-connected mixed-bed columns (1 × PLgel 5 μm MIXED-C Guard and 2 × PLgel 5 μm MIXED-C Analytical). A 390-MDS refractive index detector was employed for detection. The flow rate was set to 1 mL min^–1^, and the temperature was maintained between 35 and 40 °C. Calibration was performed using polystyrene (PS) standards.

### 3.5 Actuator Construction

The films on the PVA were detached from the glass substrate and placed between two circular, rigid plastic frames. The entire ensemble was placed in water to dissolve the PVA sacrificial layer. The washing step was repeated several times to effectively remove the PVA. Thereafter, it was dried in a vacuum oven. Circular electrodes made of carbon black powder, approximately 8 mm in diameter, were applied to both sides of the film and connected to a high-voltage source using aluminum strips. The actuation strain was measured optically by observing the change in diameter of the electrode area, which was captured using a digital camera. A LabView program equipped with an edge detection tool was used to accurately identify the boundary between the black electrode area and the transparent silicone film.

### 3.6 Stack Actuator Construction

A stack actuator was assembled by layering multiple single-layer actuators to form an interdigitated structure with alternating layers of dielectric films and electrodes. The elastic electrode material was produced via anionic ring-opening polymerization of a cyclic silicone monomer with cyanopropyl functional groups combined with a polar bicyclic cross-linker. Tetramethylammonium hydroxide (TMAH) was the initiator, enabling simultaneous polymerization and cross-linking, thereby forming an elastomer network in a single step. We first created a 110 μm thick dielectric film and cut it into 1 × 1.5 cm pieces. A 100 μm thick elastic electrode material was then cut into 1 × 1.0 cm pieces, resulting in five samples for the dielectric layers and six for the electrodes. Five dielectric layers were stacked alternatively, with the electrode layers carefully placed on each to ensure an active area of 1 cm^2^. The device was placed on a 100 °C hot plate for 1 h and then kept overnight in a vacuum oven at 60 °C. The overall thickness of the device was 1150 μm. The thickness change of the stack actuator during operation was precisely monitored using an optoNCDT 2300 laser.

## 4 Conclusions

This work represents a further development of polysiloxanes tailored for high-performance actuation systems for DE applications. Polysiloxane was modified with different 2-(ethylsulfonyl)­ethane contents, ranging from 25 to 100 mol %. This chemical modification led to polysiloxanes whose *T*_g_ increased with increasing polarity. The polysiloxanes with the highest 2-(ethylsulfonyl)­ethane content exhibited a remarkable dielectric permittivity of 16.2 at 10 kHz (25 °C) and a *T*_g_ of −27.86 °C, a value well below room temperature, which is attractive for applications at room temperatures. Such a *T*_g_ ensured flexibility and operational stability under ambient conditions, a critical requirement for practical applications. The polymers were cross-linked using pentaerythritol tetrakis­(3-mercaptopropionate), yielding elastomers with outstanding elasticity and significant strain at break. These superior material properties translated directly into exceptional actuation performance, as demonstrated by a single-layer actuator that achieved a large actuation strain of 13% under a low electric field of 8.2 V μm^–1^ (1 Hz, 900 V). Additionally, a multilayer stack actuator, comprising five dielectric layers, exhibited a thickness actuation strain of 4.5% at 14.5 V μm^–1^ and maintained stable actuation across various frequencies, including 5 and 10 Hz at 1200 V. Remarkably, the stack actuator also demonstrated reliable performance over 4000 actuation cycles at 1 Hz, showcasing its durability for long-term operation. Future work will focus on making this material solvent-free, allowing layer-by-layer manufacture of stacked actuators with dielectric thicknesses below 20 μm.

## Supplementary Material



## Notes

GPC measurements, NMR spectra of the thiols, ^13^C NMR spectra of the polymers, FTIR spectra, temperature-dependent impedance measurement of the materials, and stack actuator manufacturing details (PDF)
