# Supplementary material for: Single-Layer and Stack Dielectric Elastomer Actuators Using Polysiloxanes Modified with Ethylsulfonyl Groups
Source: ACS Appl Mater Interfaces. 2025 Jun 17;17(26):38504–14. doi: 10.1021/acsami.5c06610 (PMC12232269; doi:10.1021/acsami.5c06610)
Supplement: Supplementary file 1 [file am5c06610_si_001.pdf]

Supporting Information

Single-layer and stack dielectric elastomer actuators using polysiloxanes modified with ethylsulfonyl groups

*Cansu Zeytun Karaman[a,b], Thulasinath Raman Venkatesan[a], Frank A. Nüesch[a,b], and Dorina M. Opris[a,c]**

[a]*Functional Polymers,Empa, Swiss Federal Laboratories for Materials Science and Technology (EMPA), 8600 Duebendorf, Switzerland.*

[b]*Ecole Polytechnique Federale de Lausanne(EPFL), 1015 Lausanne, Switzerland*

[c]*Department of Materials, Eidgenössische Technische Hochschule Zürich ETHZ, CH-8092 Zurich, Switzerland*

E-mail: dorina.opris@empa.ch

Table of Content

GPC……..2

NMR Spectra of the thiols 3

^{13}C NMR Spectra of the polymers 4

FTIR……...4

Temperature dependent impedance measurement of the materials 6

Stack actuator fabrication 10

References 11

GPC

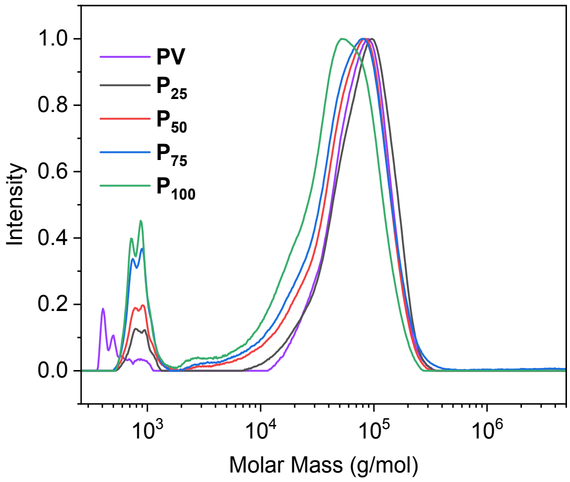

Figure S1. GPC elugrams of the polymers **PV**, $\mathbf{P_{25}}$, $\mathbf{P_{50}}$, $\mathbf{P_{75}}$, and $\mathbf{P_{100}}$ in THF using polystyrene standards.

Table S1. The molar mass and molar mass distributions results from GPC characterization.

Sample	M_n (kg mol^{-1})	M_w (kg mol^{-1})	PDI	Oligomer/cycles [wt.%]
PV	58.3	85.3	1.46	12.25
P_{25}	57.2	88.1	1.54	3.17
P_{50}	43.9	75.3	1.71	5.21
P_{75}	45.1	75.0	1.66	9.35
P_{100}	36.0	61.9	1.71	9.82

NMR Spectra of the thiols

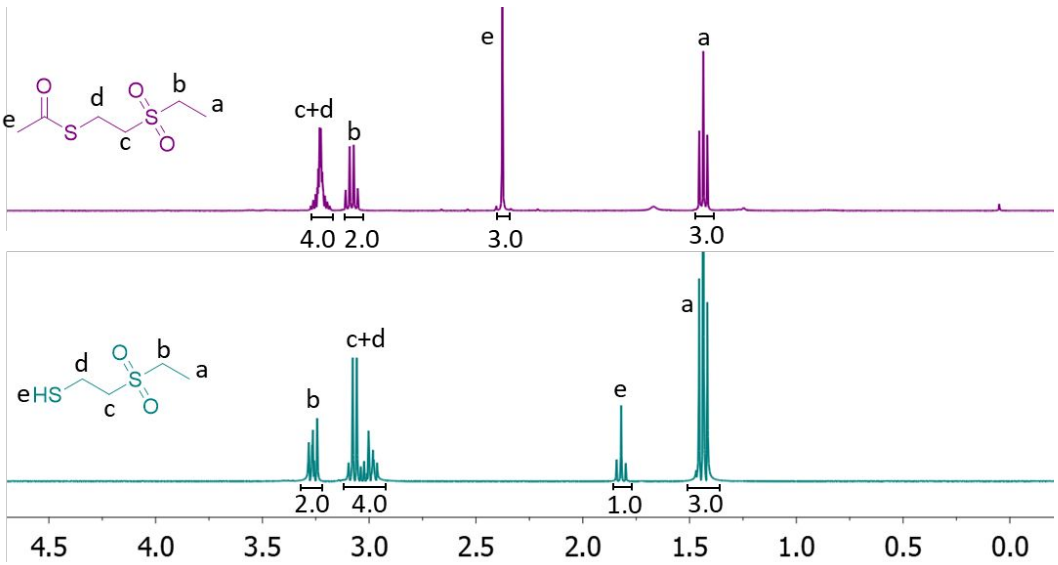

Figure S2. ^{1}H NMR spectra of the S-(2-(ethylsulfonyl)ethyl)ethanethiolate (top), and 2-(ethylsulfonyl)ethane-1-thiol (bottom) in $CDCl_3$ at room temperature.

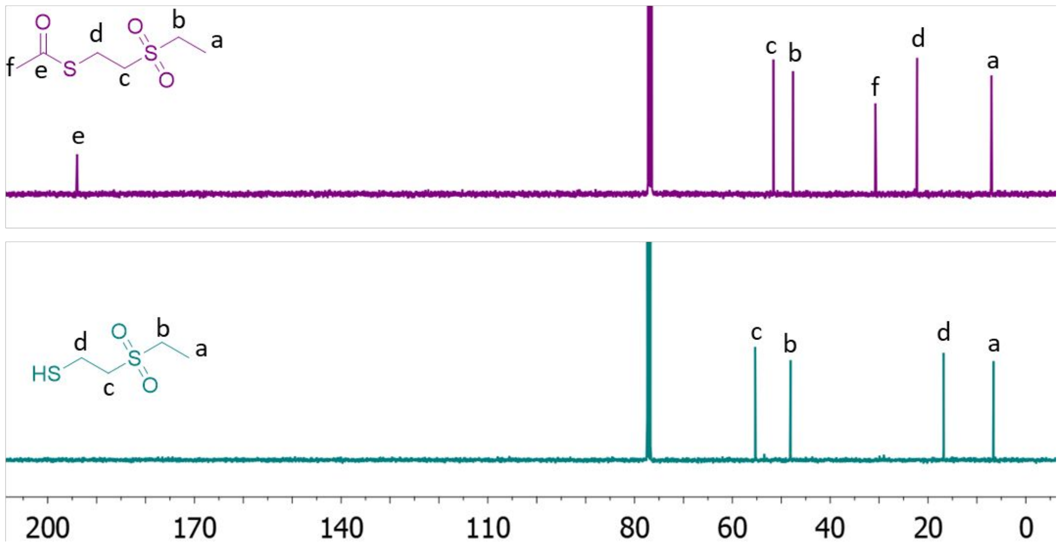

Figure S3. ^{13}C NMR spectra of the S-(2-(ethylsulfonyl)ethyl) ethanethiolate (top), and 2-(ethylsulfonyl)ethane-1-thiol (bottom) in $CDCl_3$ at room temperature.

^{13}C NMR Spectra of the polymers

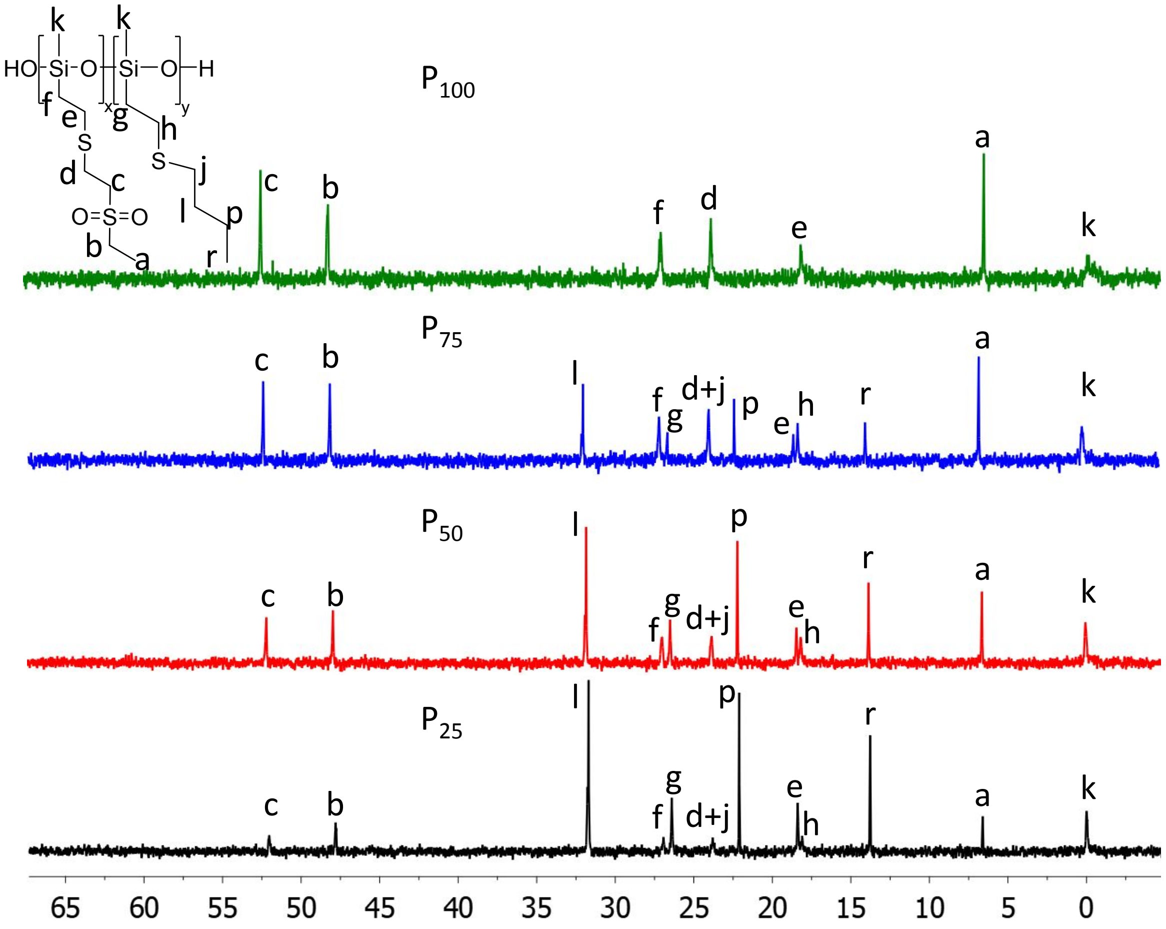

Figure S4. ^{13}C NMR spectra of the polymers **Px** in $CDCl_3$.

FTIR

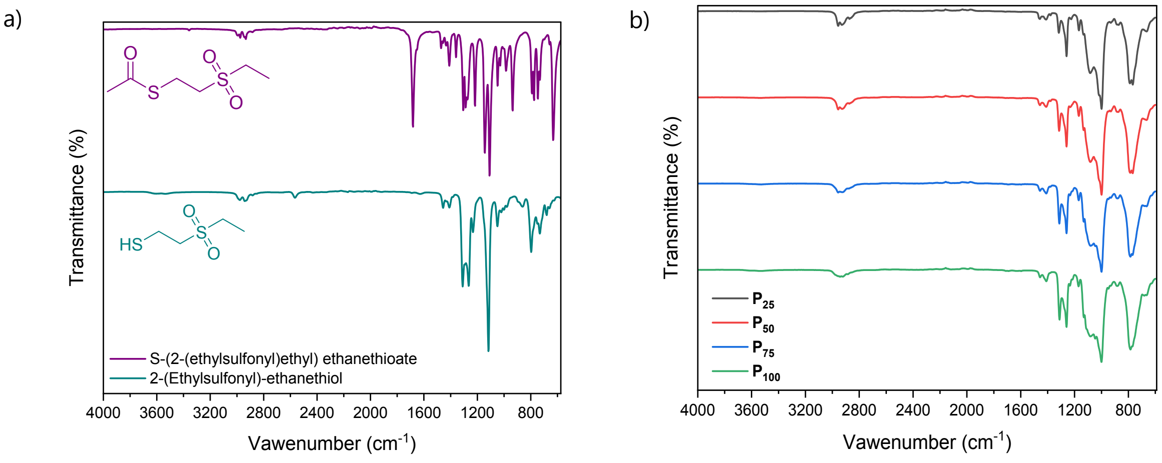

Figure S5. FTIR spectra of the S-(2-(ethylsulfonyl)ethyl) ethanethioate and 2-(ethylsulfonyl)ethane-1-thiol (a), and $\mathbf{P_x}$ (b)

FTIR is used to determine the functional groups present in the samples. In Figure S5a, the FTIR spectra of S-(2-(ethylsulfonyl)ethyl) ethanethioate and 2-(ethylsulfonyl)ethane-1-thiol are shown. Common peaks include 764 cm^{-1} (S-C), 1313 cm^{-1} (S=O), and 2933 cm^{-1} (C-H), with overlapping fingerprint peaks. However, the peak at 1682 cm^{-1} (C=O) distinguishes S-(2-(ethylsulfonyl)ethyl) ethanethioate due to the presence of the carbonyl functional group.[1]

In Figure S5b, the FTIR spectra of the materials are presented. The common peaks at 764 cm^{-1} (S-C), 1313 cm^{-1} (S=O), and 2933 cm^{-1} (C-H) are attributed to the 2-(ethylsulfonyl)ethane-1-thioether side group. Additional peaks at 1283 cm^{-1} (Si-CH_3), 1082 cm^{-1} (Si-O-Si), and 784 cm^{-1} (Si-C) characteristic of the polysiloxane backbone are observed.[2]

Temperature dependent impedance measurement of the materials

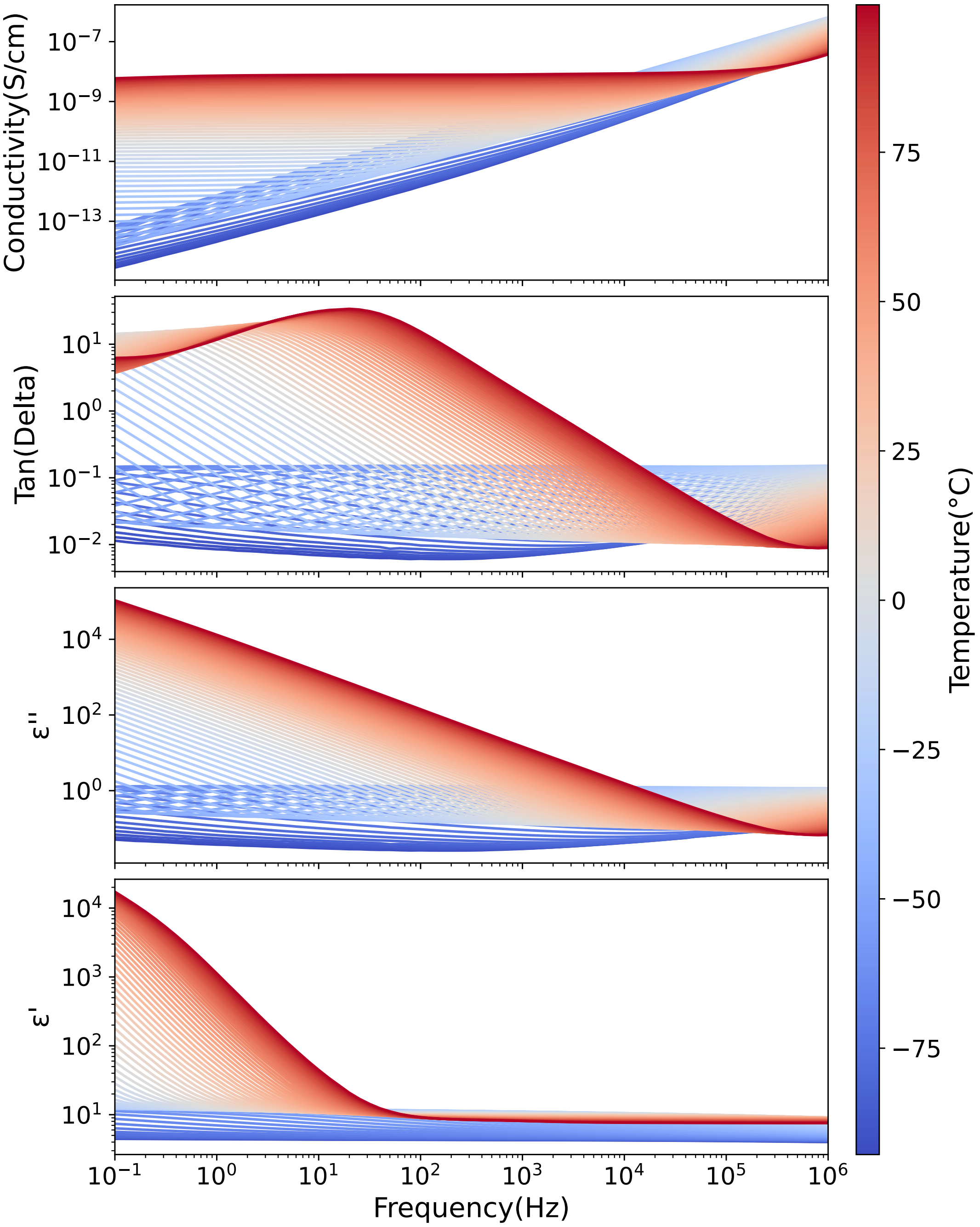

Figure S6. The conductivity (σ'), loss tangent (tan (δ)), dielectric loss (ε''), and dielectric permittivity (ε') at different temperatures as a function of frequency ranging from 10^{-1} and 10^{6} Hz for $\mathbf{M_{25}}$.

Temperature-dependent impedance measurements provide valuable insights into how dielectric permittivity (ε'), dielectric loss (ε''), tangent delta (tanδ), and conductivity vary with temperature and frequency over the range of 0.01 Hz to 1 MHz. [3,4]

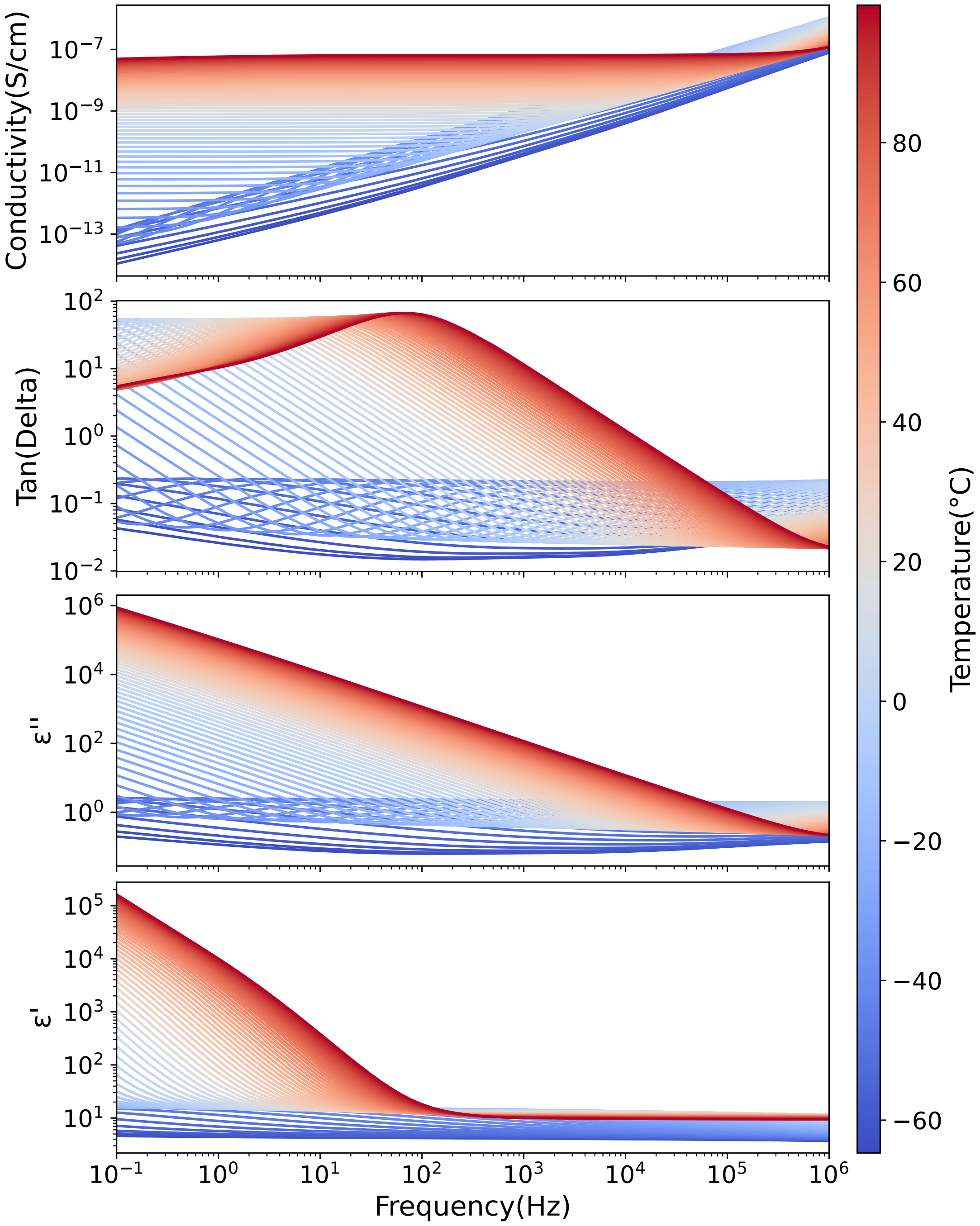

Figure S7. The conductivity (σ'), loss tangent (tan (δ)), dielectric loss (ε'') and dielectric permittivity (ε') at different temperatures as a function of frequency ranging from 10^{-1} and 10^{6} Hz for $\mathbf{M_{50}}$.

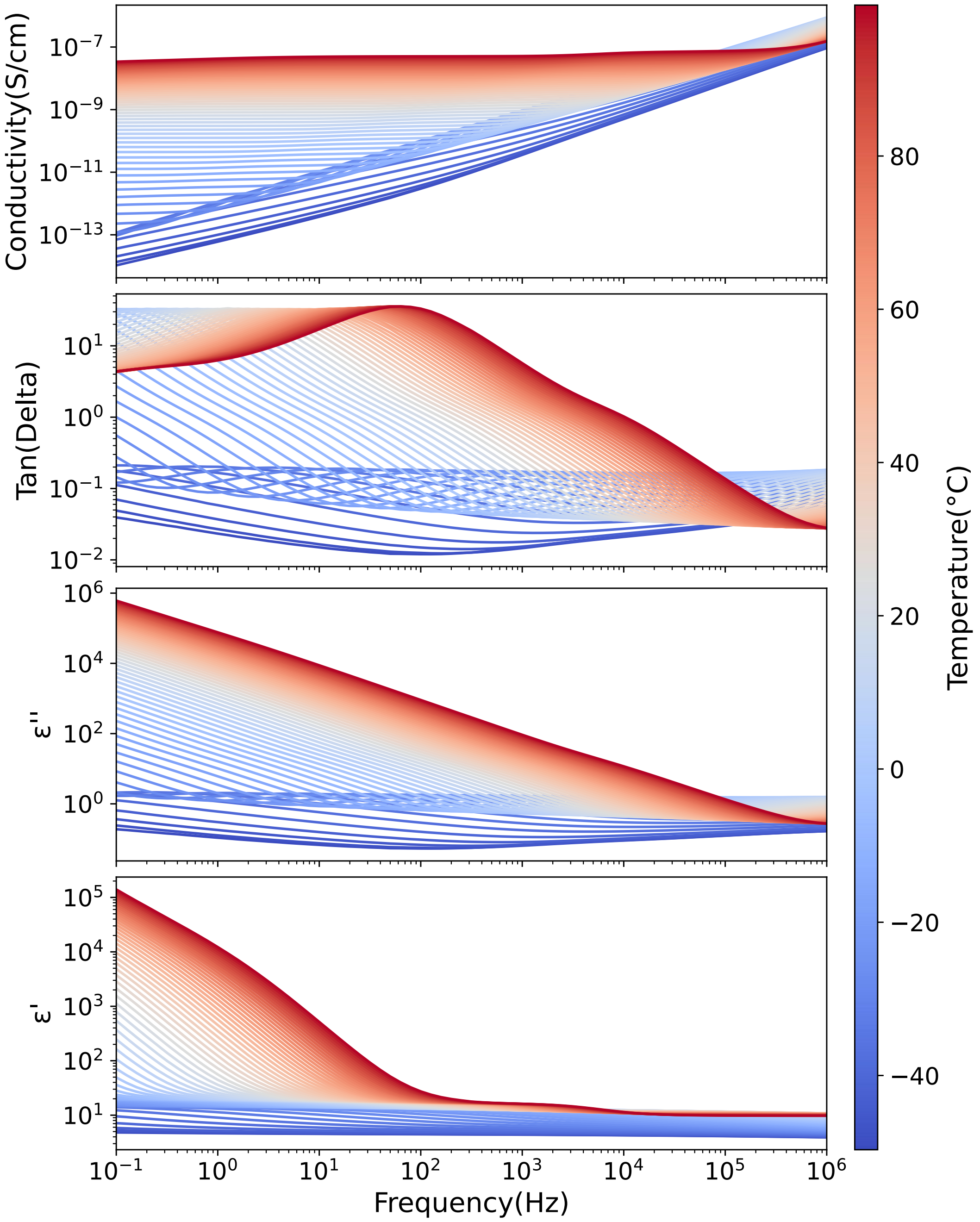

Figure S8. The conductivity (σ'), loss tangent (tan (δ)), dielectric loss (ε''), and dielectric permittivity (ε') at different temperatures as a function of frequency ranging from 10^{-1} and 10^{6} Hz for $\mathbf{M_{75}}$.

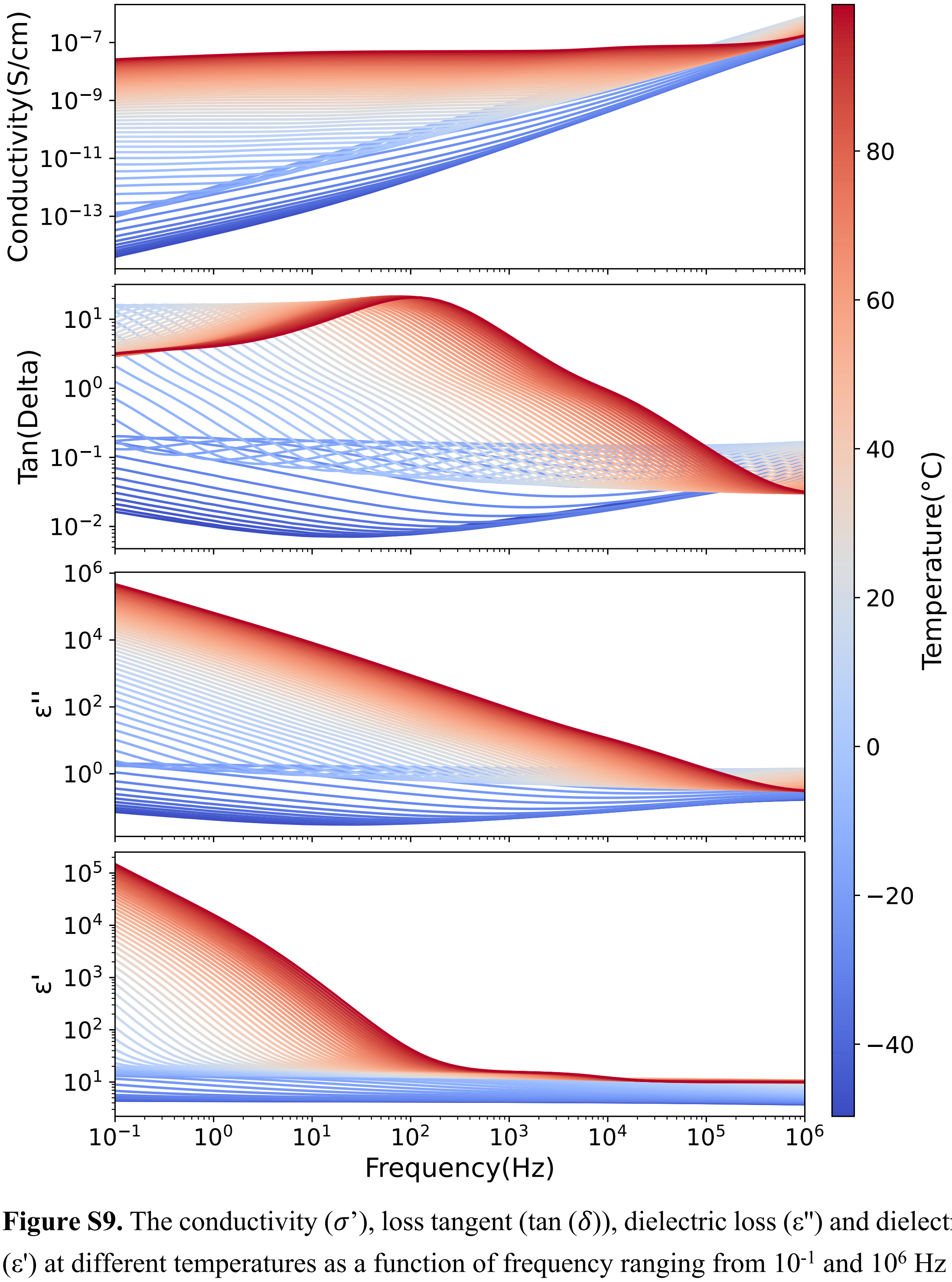

Figure S9. The conductivity (σ'), loss tangent (tan (δ)), dielectric loss (ε'') and dielectric permittivity (ε') at different temperatures as a function of frequency ranging from 10^{-1} and 10^{6} Hz for $\mathbf{M_{100}}$.

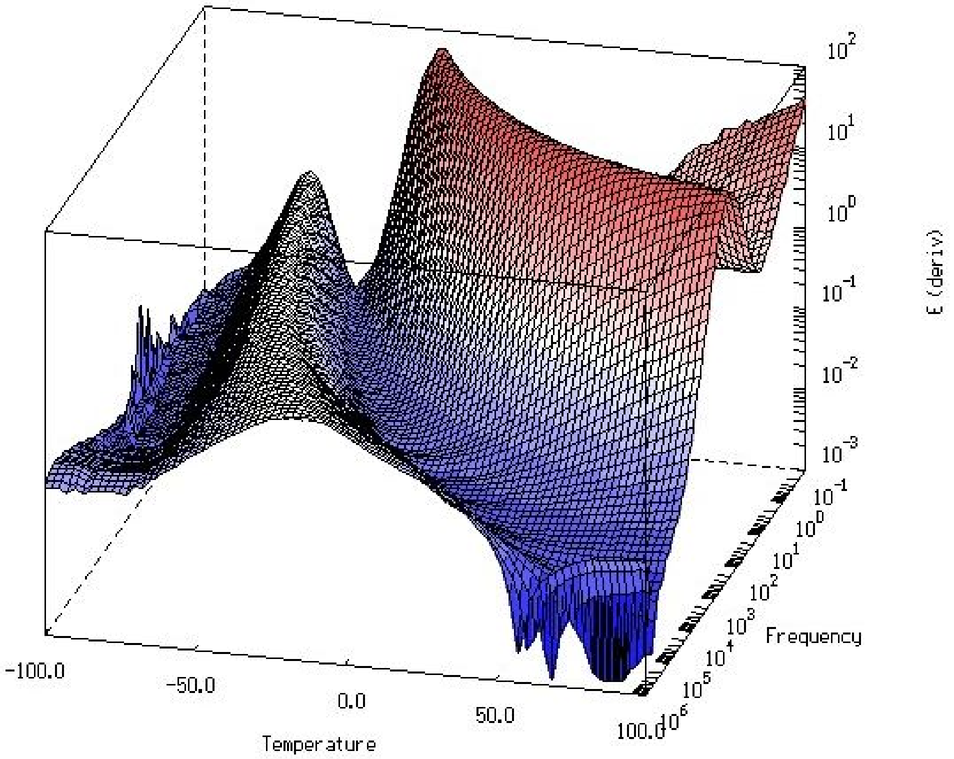

Figure S10. 3D plot of conduction-free ε″der curves of a **M_{25}** sample as a function of frequency from 10^{-1} to 10^{+6} Hz and temperatures from −100 to +100 °C.

Stack actuator fabrication

The fabrication of the stacked dielectric elastomer actuator (DEA) begins by stamping of dielectric films onto compliant electrode to form individual electrode–dielectric layer assembly. These assemblies are then carefully aligned and laminated to construct a multilayered interdigitated structure by continue to stamping several layers of altering dielectric and electrode layers, as illustrated in the schematic. This stacking process ensures proper pairing of each electrode with an adjacent dielectric layer, enabling effective charge distribution and actuation upon voltage application. To enhance interlayer adhesion and ensure structural integrity, the assembled device is heated to 100 °C, promoting bonding between layers, and subsequently placed in a vacuum oven at 60 °C overnight to remove residual solvents and improve cohesion. The final appearance of the stacked actuator is shown in the accompanying photograph.

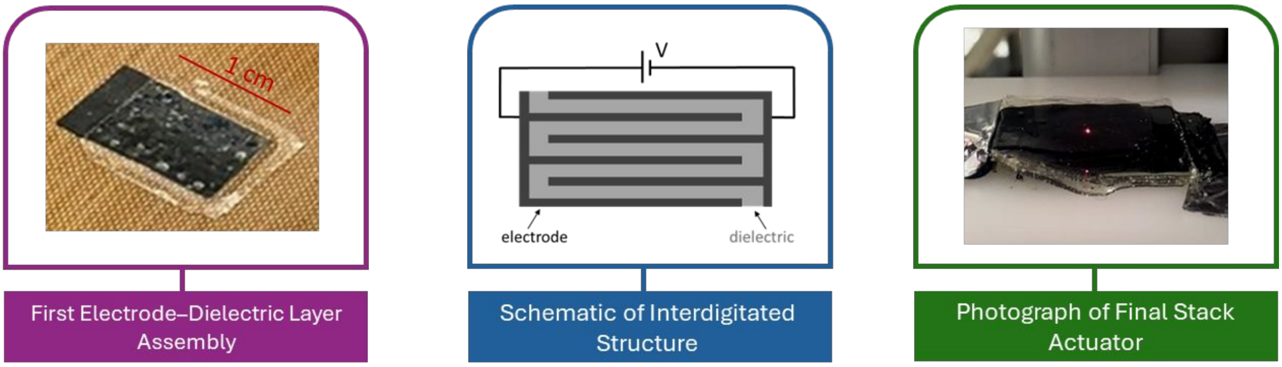

Figure S11. Schematic representation of the stack actuator construction process, including the initial electrode–dielectric layer formation (left), interdigitated stacking configuration (middle), and the final assembled actuator (right).

References

(1) Waqif, M.; Lakhdar, M.; Saur, O.; Lavalley, J. C. FTIR Study of the Influence of Sulfate Species on the Adsorption of NO, CO and NH 3 on CuO/Al 2 O 3 Catalysts. *Journal of the Chemical Society, Faraday Transactions* **1994**, *90* (18), 2815–2820. https://doi.org/10.1039/FT9949002815.

(2) Steinbach, J. C.; Schneider, M.; Hauler, O.; Lorenz, G.; Rebner, K.; Kandelbauer, A. A Process Analytical Concept for In-Line FTIR Monitoring of Polysiloxane Formation. *Polymers (Basel)* **2020**, *12* (11), 1–13. https://doi.org/10.3390/POLYM12112473.

(3) Zhao, X. Y.; Liu, H. J.; Wang, M. Z. Polymer Dielectric Materials. *Recent Advances in Dielectric Materials* **2012**, 323–368. https://doi.org/10.5772/50638.

(4) Bur, A. J. Dielectric Properties of Polymers at Microwave Frequencies: A Review. *Polymer (Guildf)* **1985**, *26* (7), 963–977. https://doi.org/10.1016/0032-3861(85)90216-2.
